# Deficiency of germinal center kinase TRAF2 and NCK-interacting kinase (TNIK) in B cells does not affect atherosclerosis

**DOI:** 10.3389/fcvm.2023.1171764

**Published:** 2023-05-05

**Authors:** Bram W. van Os, Pascal J. H. Kusters, Myrthe den Toom, Linda Beckers, Claudia M. van Tiel, Winnie G. Vos, Elize de Jong, Arnd Kieser, Cindy van Roomen, Christoph J. Binder, Myrthe E. Reiche, Menno P. de Winther, Laura A. Bosmans, Esther Lutgens

**Affiliations:** ^1^ Department of Medical Biochemistry, Amsterdam UMC Location University of Amsterdam, Amsterdam, Netherlands; ^2^ Amsterdam Cardiovascular Sciences, Atherosclerosis & Ischemic Syndromes, Amsterdam, Netherlands; ^3^ Amsterdam Immunity and Infection, Amsterdam UMC, Amsterdam, Netherlands; ^4^ Research Unit Signaling and Translation, Helmholtz Zentrum München-German Research Center for Environmental Health, Neuherberg, Germany; ^5^ Department of Laboratory Medicine, Medical University of Vienna, Vienna, Austria; ^6^ Institute for Cardiovascular Prevention (IPEK), Ludwig-Maximilians-Universität, Munich, Germany; ^7^ German Center for Cardiovascular Research (DZHK), Partner site Munich Heart Alliance, Ludwig-Maximilians-Universität München, Germany; ^8^ Department of Cardiovascular Medicine and Immunology, Mayo Clinic, Rochester, MN, United States

**Keywords:** TNIK, B cells, signaling, atherosclerosis, IgA

## Abstract

**Background:**

Atherosclerosis is the underlying cause of many cardiovascular diseases, such as myocardial infarction or stroke. B cells, and their production of pro- and anti-atherogenic antibodies, play an important role in atherosclerosis. In B cells, TRAF2 and NCK-interacting Kinase (TNIK), a germinal center kinase, was shown to bind to TNF-receptor associated factor 6 (TRAF6), and to be involved in JNK and NF-κB signaling in human B cells, a pathway associated with antibody production.

**Objective:**

We here investigate the role of TNIK-deficient B cells in atherosclerosis.

**Results:**

*ApoE^−/−^TNIK^fl/fl^* (*TNIK^BWT^*) and *ApoE^−/−^TNIK^fl/fl^CD19-cre* (*TNIK^BKO^*) mice received a high cholesterol diet for 10 weeks. Atherosclerotic plaque area did not differ between *TNIK^BKO^* and *TNIK^BWT^* mice, nor was there any difference in plaque necrotic core, macrophage, T cell, α-SMA and collagen content. B1 and B2 cell numbers did not change in *TNIK^BKO^* mice, and marginal zone, follicular or germinal center B cells were unaffected. Total IgM and IgG levels, as well as oxidation specific epitope (OSE) IgM and IgG levels, did not change in absence of B cell TNIK. In contrast, plasma IgA levels were decreased in *TNIK^BKO^* mice, whereas the number of IgA^+^ B cells in intestinal Peyer's patches increased. No effects could be detected on T cell or myeloid cell numbers or subsets.

**Conclusion:**

We here conclude that in hyperlipidemic *ApoE^−/−^* mice, B cell specific TNIK deficiency does not affect atherosclerosis.

## 1. Introduction

Cardiovascular disease (CVD) is the leading cause of morbidity and mortality world-wide, with one in four deaths being linked to CVD (1, 2). Atherosclerosis (AS) is the most frequent underlying pathology of CVD, causing clinical features, such as myocardial infarction, stroke or heart failure. In recent years AS has been recognized as a chronic inflammatory lipid-driven disease of mid-sized and large arteries, characterized by the formation of lumen encroaching plaques (3, 4).

B cells are an important player in the adaptive immune system and various B cell subsets have different roles in the initiation and progression of atherosclerosis. Two distinct B cell subsets are identified, the innate like B1 cell and the conventional B2 subset (5). B1 B cells are recognized for their anti-atherogenic properties through the production of protective immunoglobulin (Ig)M antibodies, reactive against oxidation specific epitopes (OSE) (6). Most B2 cell subsets, but not marginal zone B cells, and plasma cells are considered pro-atherogenic, as their deficiency reduces atherosclerosis (7). Consequently, B cell depletion in atherosclerotic mice via an anti-CD20 antibody, reduced cardiovascular burden (8, 9).

The TRAF2 and NCK-interacting Kinase (TNIK) is a ubiquitously expressed member of the germinal center kinase family (10). In B cells, TNIK was identified as an interaction partner of the latent membrane protein (LMP1) signalosome after EBV infection, as well as of LMP1's cellular counterpart, the immune checkpoint CD40. Specifically, TNIK was reported to directly bind TRAF6, thereby bridging its interaction with the C-terminus of LMP1/CD40. In this complex, TNIK's C-terminal kinase part is essential for JNK activation, whereas its N-terminal part induces IKKβ/NF-κB activation, both essential for B cell activation (11).

As a regulatory component of the Wnt signaling pathways β-catenin and T-cell factor-4 (TCF-4) complex, TNIK displays divergent functions in different cell types (12). Studies reveal that TNIK is essential for the activation of Wnt target genes, allowing for proliferation of tumor cells (13), which induces tumor progression of myelogenous leukemia, colorectal cancer and lung non-small cell carcinoma (14–16). Similar results were observed in CD8^+^ TNIK deficient T cells, as these cells lost the capability to expand and have impeded memory formation, caused by TNIK-CD27-mediated activation of the Wnt signaling pathway (17).

In our previous research, we have shown that whole body deficiency of CD40, as well as cell-specific deficiency of CD40 in dendritic cells, adipocytes and macrophages, reduces atherosclerosis and especially plaque inflammation (18–21). We have also shown a pivotal role for the macrophage CD40-TRAF6-NF-kB pathway in driving atherosclerosis (22). Moreover, mixed chimeric *Ldlr*^−/−^ mice whose B cells are deficient in CD40 show a reduction in atherosclerosis (23). As TNIK seems to be involved in the CD40-TRAF6 signaling complex, and as B cell CD40 activation seems to accelerate atherosclerosis, we here aimed to elucidate the role of TNIK signaling in B cells in atherosclerosis.

## 2. Materials and methods

### 2.1. Mice

*ApoE^−/−^TNIK^fl/fl^* (*TNIK^BWT^*) and *ApoE^−/−^TNIK^fl/fl^ CD19-cre* (*TNIK^BKO^*) mice were generated and bred at the animal facility of the Amsterdam University Medical Centers, University of Amsterdam, the Netherlands. Eight-week-old female *TNIK^BWT^* and *TNIK^BKO^* littermates received a high cholesterol diet, containing 0.15% cholesterol (Arie Blok Animal Nutrition, Woerden, NL) for 10 weeks. The mice had access to food and water *ad libitum* and were housed according to institutional guidelines.

Mice were humanely killed by a combination of carbon dioxide and exsanguination as blood samples were collected via intracardiac puncture in ethylenediaminetetraacetic acid (EDTA)-coated syringes. Mice were perfused with phosphate buffered saline (PBS) to reduce any blood contamination and organs were harvested for analysis. All animal experiments were conducted at the Amsterdam University Medical Center, location Academic Medical Center, University of Amsterdam, NL and approved by the committee for animal welfare and the Dutch Centrale Commissie Proefdieren (AVD1180020171666).

### 2.2. TNIK knockout efficiency and NF-κB signaling

B cells were isolated by magnetic bead isolation with mouse Pan B Cell Isolation beads (Miltenyi Biotec, #130-095-813) using the manufacturers provided protocol. Isolated B cells were exposed to 10 µg/ml αCD40 (BioXCell, #BE0016-2) for 4 h. Proteins were measured by western blot by staining with αTNIK (Abcam, ab95887), αNF-κB p65 (cell Signaling, #3033) and αPhospho-NF-κB p65 (Cell signaling, #6956). αAlpha-tubulin (Sanbio, CLT9002) or αActin (Sigma-Aldrich A3853) were used as a loading control. TNIK and NF-κB p65 were visualized by staining with HRP conjugated Goat anti-Rabbit (Invitrogen, #32260) and Goat anti-Mouse (Invitrogen, #32330). Phospho-NF-κB p65 and alpha-tubulin were staining with IRDye 800CW conjugated Goat anti-Mouse (Odyssey, #926-32210) and Goat anti-Rabbit IRDye 680RD (Odyssey, #926-68071). Data was analyzed using FiJi.

### 2.3. Histology

Isolated aortic arches were fixed in 1% paraformaldehyde and embedded in paraffin. To analyze plaque size in the aortic arch, the arch was cut in 4 µm sections, where every 5th section was stained with hematoxylin and eosin (H&E; Merck, Kenilworth, USA and Klinipath/VWR International, Radnor, USA).

Plaque collagen content was visualized by picro Sirius Red staining (Sigma-Aldrich, Saint Louis, Missouri, USA). Immunohistochemical staining for alpha smooth muscle actin (α-SMA; 1:3,000; Sigma-Aldrich), macrophages (Mac-3; 1:100, BD Pharmingen) and CD3^+^ T cells (CD3, 1:100, AbD Serotec) were applied and counterstaining was performed with hematoxylin.

Images were taken using a Leica CRT6 with LAS X software and analyzed using Adobe Photoshop (2021), where positively stained area (for Sirius Red, Mac-3 and α-SMA) were quantified using color threshold measurements while cells positive for CD3 were counted by a researcher blinded for the experimental conditions. Necrotic regions were defined as plaque regions negative for cells or stromal tissue.

### 2.4. Plasma lipid levels

Blood was isolated and spun down (2,100 rpm, 10 min at 4°C), and plasma was collected. Total cholesterol and triglycerides were measured by standard enzymatic methods according to manufacturer's instructions (CHOD and GPO BIOLABO).

### 2.5. Immunoglobulins

Blood was obtained via intracardial puncture and spun down (2,100 rpm, 10 min at 4°C). Total IgM, IgA and IgG concentrations of plasma were detected using Enzyme-Linked Immuno Sorbent Assay (ELISA), IgG, IgA and IgM (total) mouse uncoated ELISA kits (ThermoFisher). IgM and IgG antibodies specific to CuOx-LDL and MDA-LDL and reactivity of the AB1-2 antibody (against the T15/E06 anti-idiotype) were determined as described previously (24).

### 2.6. *In vitro* generation of IgG and IgM isotype switched B cells

Naive B cells were isolated from Peyer's patches and spleen using magnetic CD43^+^ bead isolation (Miltenyi Biotec, #130-049-801) following the manufacturer's provided protocol. To generate IgG producing plasma cells, naive B cells were exposed to 50 µg/ml LPS (InvivoGen, tlrl-pb5lps) and 10 ng/ml IL-4 (Peprotech, #214-14) for 3 days. To generate IgA producing plasma cells, naive B cells were exposed to 20 μg/ml αIgD (ThermoFisher scientific, 16-5924), 5 ng/ml IL-4 (Peprotech, #214-14), 1 ng/ml IL-5 (R&D systems, 405-ML), 10 ng/ml TGF-β (R&D systems, 7666-MB) and 20 μg/ml αCD40 (BioXcell, BE0016-2). Cells were stained in FACS buffer (0.5% bovine serum albumin, 5 mM EDTA in PBS; pH 7.4) with the following antibodies: αCD3 (1:100, APC, Biolegend, #100312), αCD11b (1:100, APC, BD Biosciences, #553312), αCD45 (1:100, APC-Cy7, Biolegend, #103115), αIgA (1:100, PE, ThermoFisher scientific, #12-4201), αIgG (1:200, FITC, Biolegend, #406001), αIgM (1:1,000, PE-Cy7, ThermoFisher scientific, #25-5790).

### 2.7. Flow cytometry

Single cell suspensions of lymph nodes, Peyer's patches and spleens were prepared by crushing the tissue through a 70 µm cell strainer (Corning). Erythrocytes in blood and spleen were removed by incubation with hypotonic lysis buffer (HLB) (160 mM ammonium chloride, 10 mM sodium bicarbonate, 1.3 mM EDTA; pH 7.4). Cells were washed with PBS and resuspended and stained in FACS buffer. Gating strategy can be found in Supplementary Methods.

To identify pre-B cells and discriminate between follicular and marginal zone cells, the following antibodies were used: αB220 (1:200, APC-eFluor780, ThermoFisher Scientific, #47-0452), αCD16/CD32 (1:1,000, Biolegend, #101330), αCD19 (1:200, PE, ThermoFisher Scientific, #12-0193), αCD21 (1:100, BV421, BD Biosciences, #562756), αCD23 (1:100, BV510, BD biosciences, #563200), αCD38 (1:100, FITC, BD Biosciences, #558813), αCD93 (1:100, APC, ThermoFisher scientific, #17-5892), αIgM (1:1600, PE-Cy7, ThermoFisher scientific, #25-5790), Prior to analysis 7-AAD (final concentration of 1 μg/ml, ThermoFisher scientific, A1310) was added to exclude dead cells.

To identify germinal center and plasma cells, the following antibodies were used: αB220 (1:200, APC-eFluor780, ThermoFisher Scientific, #47-0452), αCD16/CD32 (1:1,000, Biolegend, #101330), αCD19 (1:200, PE, ThermoFisher Scientific, #12-0193), αCD23 (1:100, BV510, BD biosciences, #563200), αCD95 (1:100, AF647, BD biosciences, #563647), αCD138 (1:100, BV421, Biolegend, #562610), αGL7 (1:100, AF488, ThermoFisher scientific, #53-5902), αIgM (1:1,600, PE-Cy7, ThermoFisher scientific, #25-5790), Prior to analysis 7-AAD (final concentration of 1μg/ml, ThermoFisher scientific, A1310) was added to exclude dead cells.

Identification of T cell naive and memory populations, the following antibodies were used: αCD3 (1:200, APC-Cy7, Biolegend, #100222), αCD4 (1:1,000, BV650, Biolegend, #100469), αCD8 (1:400, BV605, Biolegend, #100744), αCD16/CD32 (1:1,000, Biolegend, #101330), αCD44 (1:800, FITC, Biolegend, #103006), αCD62l (1:1,000, PE-Cy7, Biolegend, 104418). Prior to analysis 7-AAD (final concentration of 1μg/ml, ThermoFisher scientific, A1310) was added to exclude dead cells.

Follicular T cells and dendritic cells, were identified using the following antibodies: αCD3 (1:300, FITC, Biolegend, #100306), αCD4 (1:1,000, APC, Biolegend, #100516), αCD11c (1:100, BV421, ThermoFisher scientific, #48-0114), αCD16/CD32 (1:1,000, Biolegend, #101330), αCD23 (1:100, BV510, BD biosciences, #563200), αCXCR5 (1:100, PE-Cy7, Biolegend, 145516), αPD-1 (1:100, PE, Biolegend, 109104). Prior to analysis 7-AAD (final concentration of 1μg/ml, ThermoFisher scientific, A1310) was added to exclude dead cells.

Populations from the myeloid lineage and their activation status was identified using the following antibodies: αCD11b (1:200, BV711, Biolegend, #101241), αCD11c (1:100, PE-Cy7, ThermoFisher scientific, #25-0114), αCD16/CD32 (1:1,000, Biolegend, #101330), αCD40 (1:100, PE, Biolegend, #124609), αCD45 (1:100, APC-Cy7, Biolegend, #103115), αCD86 (1:100, BV650, Biolegend, #105035), αLy6C (1:800, AF647, Biolegend, #128010), αLy6G (1:200, FITC, ThermoFisher scientific, #11-5931), αMHC class II (1:200, BV510, Biolegend, #107635), αSiglec- F (1:100, BV421, BD Biosciences, #562681).

Bone marrow was isolated by gently scraping away the bone at the joint and flushing ice-cold PBS through. Samples were incubated 5 min on ice in HLB followed by a PBS washing step. Samples were resuspended in FACS buffer. To identify stem cell populations, samples were stained in FACS buffer with the following antibodies: αCD16/32 (1:50, BV711, Biolegend, #101337), αCD27 (1:100, BUV395, BD Biosciences, #740247), αCD34 (1:50, eFluor450, ThermoFisher scientific, #48-0341), αCD48 (1:100, APC-Cy7, Biolegend, #103432), αCD127 (1:50, PE-Cy7, ThermoFisher scientific #25-1271), αCD135 (1:100, APC, Biolegend, #135310), αCD150 (1:100, PerCP-eFluor710, ThermoFisher scientific, #46-1502), αc-kit (1:100, PE, Biolegend, #105808), αlineage cocktail (1:5, FITC, ThermoFisher scientific, #22-7770) and αSca-1 (1:100, V500, BD Biosciences, #561228). Prior to analysis DAPI (final concentration of 15 ng/ml, ThermoFisher scientific, D21490) was added to exclude dead cells.

To identify mature cells that returned to the bone marrow, samples were stained in FACS buffer with the following antibodies: αCD3 (1:200, APC, Biolegend, #100312), αCD4 (1:400, BV650, Biolegend, #100469), αCD8 (1:400, BV605, Biolegend, #100744), αCD16/CD32 (1:1,000, Biolegend, #101330), αCD19 (1:100, PerCP-Cy5.5, ThermoFisher scientific, #45-0193), αCD44 (1:300, FITC, Biolegend, #103006), αCD45 (1:100, APC-Cy7, Biolegend, #103115), αCD62l (1:800, PE-Cy7, Biolegend, 104418), αCD138 (1:200, BV421, Biolegend, #562610), αc-kit (1:100, PE, Biolegend, #105808). Prior to analysis DAPI (final concentration of 15 ng/ml, ThermoFisher scientific, D21490) was added to exclude dead cells.

Cells were measured on a LSRFortessa Cell Analyzer (BD Biosciences) or a Symphony A1 Cell analyzer (BD Biosciences) and analyzed using FCS Express software, version 7 (De Novo Software).

### 2.8. Statistics

Data are presented as mean ± standard deviation, unless stated otherwise. Statistical analyses were performed using GraphPad Prism 9.3.1 software. Data was analyzed with an unpaired t-test or unpaired non-parametric Mann-Whitney U test depending on data distribution. Two-way ANOVA was used where appropriate, and outliers were identified using Grubbs test (*α* = 0.05). *p ≤ 0.05, **p ≤ 0.01.

## 3. Results

### 3.1. B cell specific TNIK deficiency does not affect atherosclerosis

To determine the role of B cell TNIK in atherosclerosis, we generated *ApoE^−/−^TNIK^fl/fl^* (*TNIK^BWT^*) and *ApoE^−/−^TNIK^fl/fl^ CD19-cre* (TNIK^BKO^) mice, which were fed a high cholesterol diet for 10 weeks. The mice had a knock-down efficiency of B-cell TNIK of ∼90% (Supplementary Figure S1).

**Figure 1:**
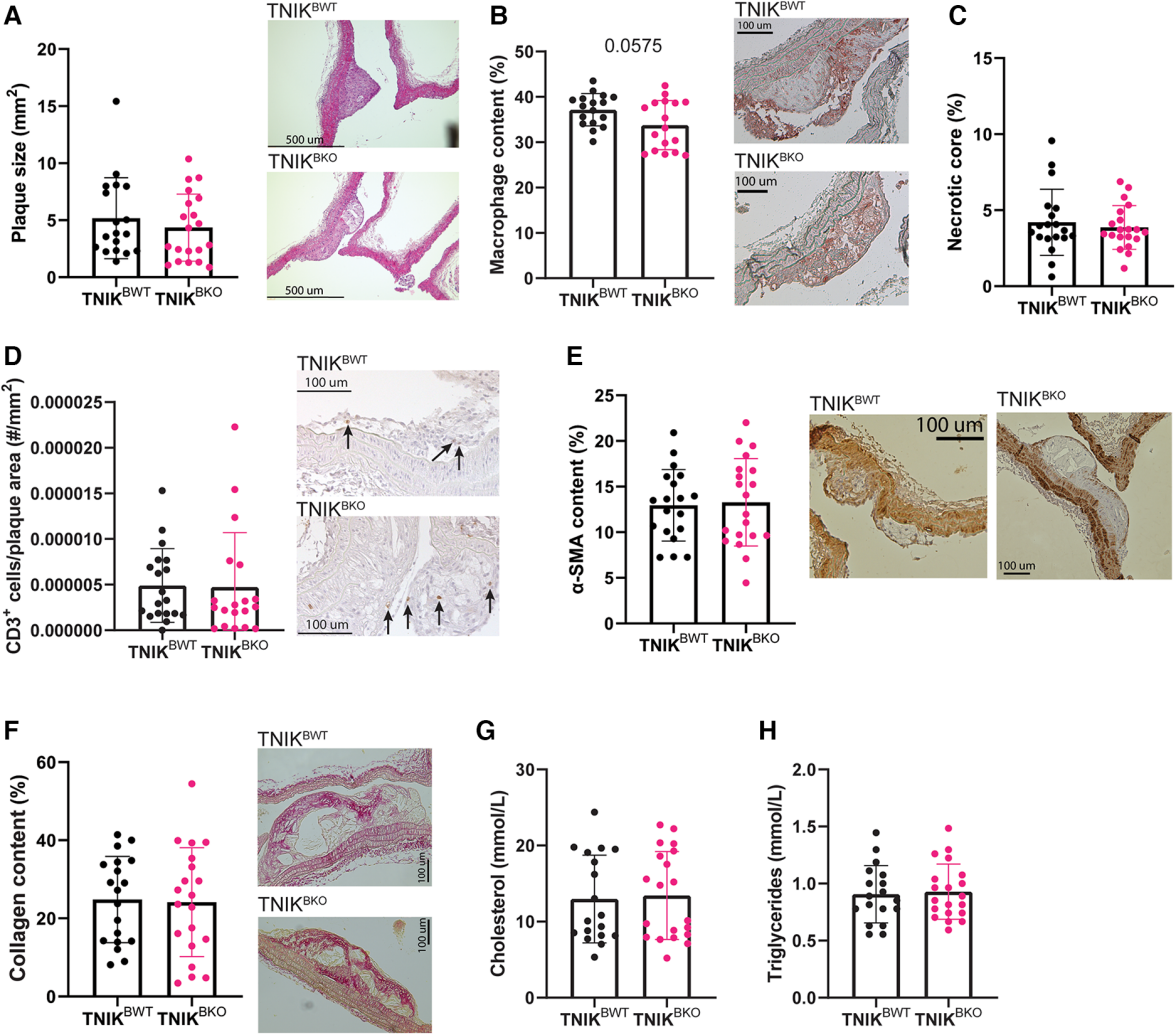
B cell TNIK deficiency does not alter plaque size, nor composition. Aortic arches of *ApoE^−/−^TNIK^flf^* (TNIK^BWT^) and *ApoE^−/−^TNIK^flfl^CD19-cre* (TNIK^BKO^) mice were isolated and analyzed for (**A**) atherosclerotic plaque size (*n* = 19/20) and (**B–F**) plaque composition. Representative images are shown. (**B**) Necrotic core content as percentage of plaque area (*n* = 19/20), (**C**) Mac3^+^ macrophage content (*n* = 17/17), (**D**) CD3^+^ T cells in plaques (*n* = 19/19), (**E**) α-SMA^+^ smooth muscle cells (*n* = 19/20) and (**F**) collagen content (*n* = 19/20) did not differ between *TNIK^BWT^* and *TNIK^BKO^* mice. (**G**) Plasma cholesterol (*n* = 18/20) and (**H**) triglyceride (*n* = 19/20) concentrations were unaltered in *TNIK^BWT^* and *TNIK^BKO^* mice.

Atherosclerotic plaque area in the aortic arch and its main branch points did not differ between *TNIK^BWT^* and *TNIK^BKO^* mice (Figure 1A). Similar results were found in the aortic root (Supplementary Figure S2). Apart from a trend towards a decrease in macrophage content in *TNIK^BKO^* mice (Figure 1B), other plaque composition features, including necrotic core content (Figure 1C), CD3^+^ T cell content (Figure 1D), α-SMA^+^ smooth muscle cell content (Figure 1E) and collagen content (Figure 1F), were not affected by B cell specific TNIK deficiency, as were plasma cholesterol and triglyceride levels (Figures 1G–H). These data indicate that B cell specific TNIK deletion does not affect atherosclerotic lesion development or composition.

### 3.2. TNIK deletion does not alter B cell subsets and function

**Figure 2:**
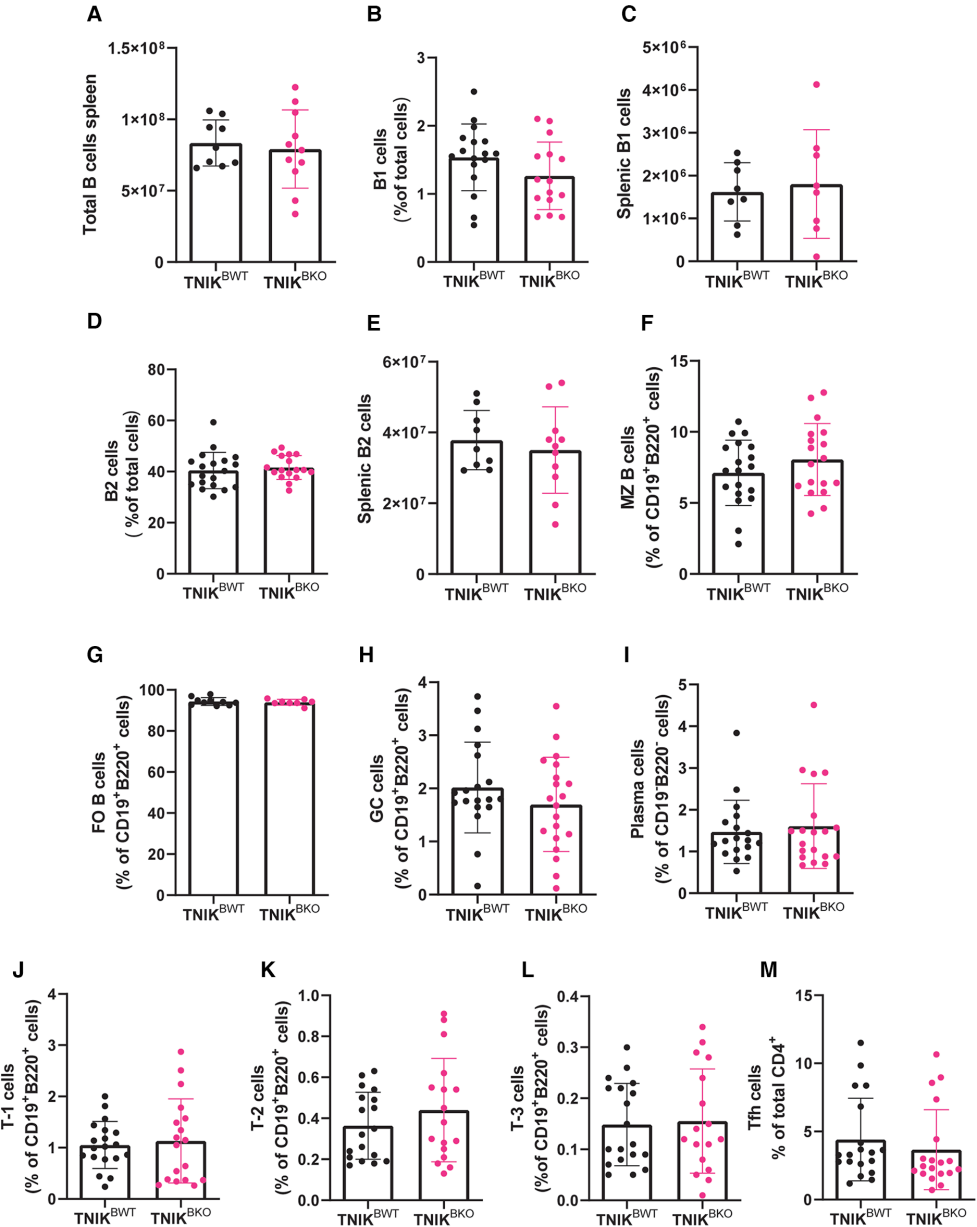
Splenic B cell differentiation and subsets unaltered due to B cell specific TNIK deficiency in atherogenic mice. The differentiation and subsets of B cells are measured by flow cytometry in the spleen of *TNIK^BWT^* and *TNIK^BKO^* mice. (**A**) The total number of splenic B cells (CD19^+^B220^+^) are similar between *TNIK^BWT^* and *TNIK^BKO^* mice (n = 9/11). The (**B**) relative fraction of splenic B1 cells (CD19^low^B220^+^) (n = 17/15), (**C**) total splenic B1 cell (n = 8/8), (**D**) relative number of B2 cells (CD19^+^B220^+^) (n = 19/17), (**E**) and total splenic B2 cells (n = 9/11)are unaffected in *TNIK^BKO^* compared to *TNIK^BWT^* mice. Further subdivision of B2 cells into (**F**) marginal zone (CD19^+^B220^+^CD21^+^CD23^−^) (n = 19/18), (**G**) follicular (CD19^+^B220^+^CD21^low^CD23^+^) (n = 19/18) and (**H**) germinal center B cells (CD19^+^B220^+^CD95^+^GL7^+^) (n = 19/20), as well as (**I**) plasma cells (CD19^−^B220^−^CD238^+^) (n = 18/19) showed no differences between genotypes. Transitional stage (CD19^+^B220^+^CD93^+^) (**J**) T-1 (IgM^+^CD23^−^) (n = 19/18), (**K**) T-2 (IgM^+^CD23^+^) (n = 18/17), and (**L**) T-3 (IgM^low^CD23^+^) (n = 19/17) B cells were similar between *TNIK^BWT^* and *TNIK^BKO^* mice. (**M**) Follicular helper T cells (CD3^+^CD4^+^CXCR5^+^PD-1^+^) are also unchanged between *TNIK^BWT^* and *TNIK^BKO^* mice (n = 19/19).

Next, we examined the effects of B cell TNIK deficiency on composition of B cell subsets in spleen (Figure 2) and blood (Supplementary Figures S3A–I). The total number of splenic B cells and of B1 and B2 B cells in spleens was similar in *TNIK^BKO^* and *TNIK^BWT^* mice (Figures 2A–E), as was the fraction of marginal zone (MZ), follicular (FO), germinal center (GC) B cells, and plasma cells (Figures 2F–I). Splenic transitional stage T-1, T-2 and T-3 B cells were also unaltered (Figures 2J–L). Moreover, the fraction of follicular helper T cells (Tfh), associated with germinal center cell transition, was similar in *TNIK^BWT^* and *TNIK^BKO^* mice (Figure 2M). Our analyses in blood phenocopied those from spleen (Supplementary Figures 3A–I).

**Figure 3:**
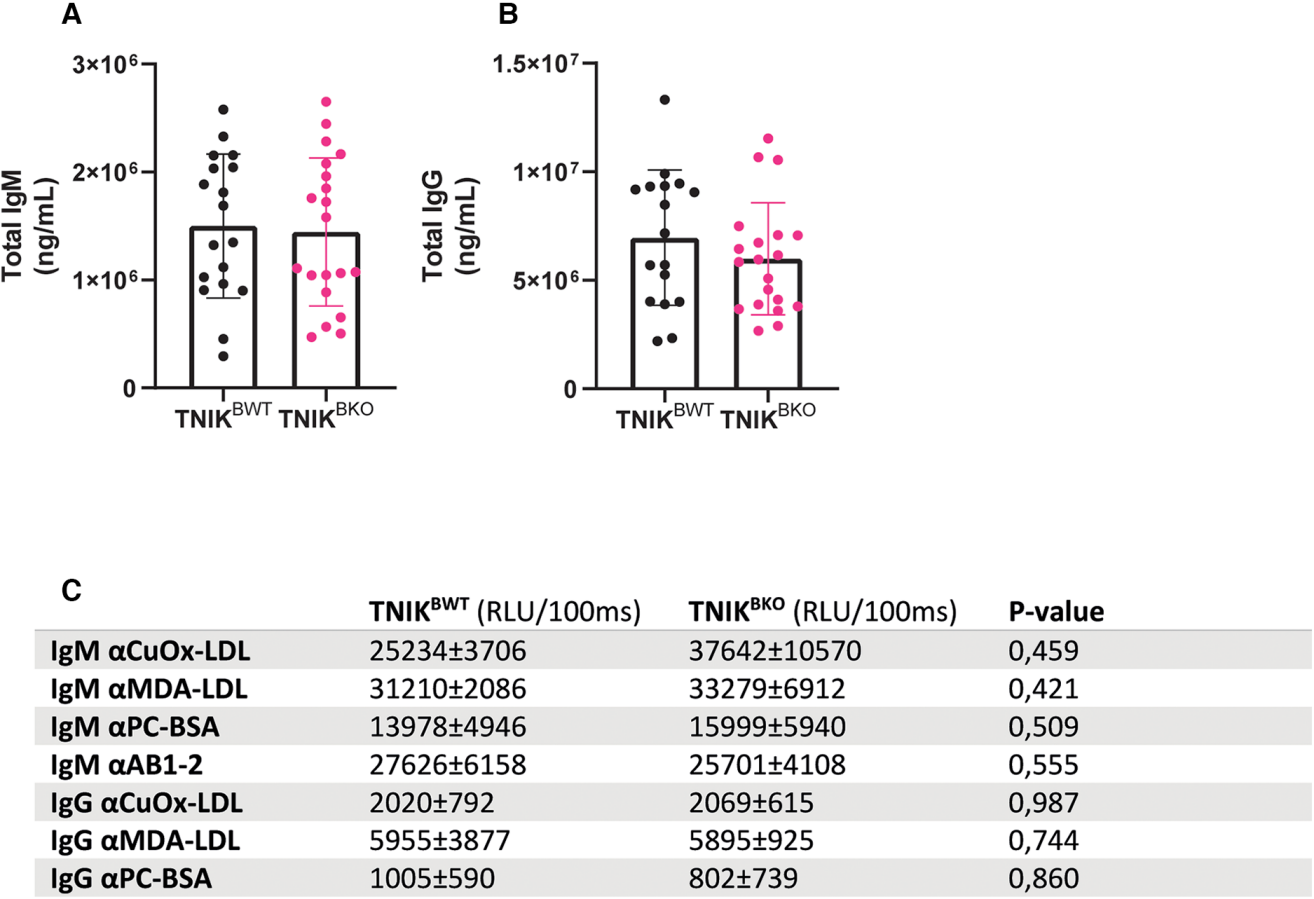
Plasma levels of IgM and IgG and anti-oxLDL immunoglobulin levels are unaffected due to B cell specific TNIK deficiency. Blood from female *TNIK^BWT^* and *TNIK^BKO^* mice, fed a high cholesterol diet for 10 weeks, was analyzed for plasma levels of total IgM and IgG and their lipid oxidation-specific epitopes (OSE). (**A**) Total plasma IgM (*n* = 18/20) and (**B**) IgG levels (*n* = 17/20) were unaffected in *TNIK^BKO^* compared to *TNIK^BWT^*. Concentrations of OSE specific (**C**) IgM and IgG were similar in *TNIK^BKO^* compared to *TNIK^BWT^* (*n* = 8/8). Data expressed as relative light units per 100 ms ± SD.

Total plasma IgM and IgG levels did not differ between *TNIK^BWT^* and *TNIK^BKO^* mice (Figures 3A–B). Levels of IgM and IgG antibodies directed against oxidation-specific epitopes (OSE), including anti-copper-oxide containing low density lipoprotein (CuOx-LDL), anti-malondialdehyde-modified LDL (MDA-LDL), anti-phosphocholine (PC-BSA) and E06/T15 idiotype (AB1-2) (Figure 3C) were also similar in both genotypes.

TNIK was identified as an essential part of the Wnt signaling pathway (12) and it was shown to also activate NF-κB via TRAF2, an important mediator in B cell signaling (25). Furthermore, it has been suggested that TNIK could signal via TRAF6 (11). Yet, when we activate CD40, no differences were observed in protein levels of NF-κB and phosphorylated NF-κB, nor in the ratio between phosphorylated NF-κB of total NF-κB in *TNIK^BKO^* mice (Supplementary Figures S4A–C).

### 3.3. Intestinal IgA is increased in B cell deficient TNIK atherosclerotic mice

**Figure 4:**
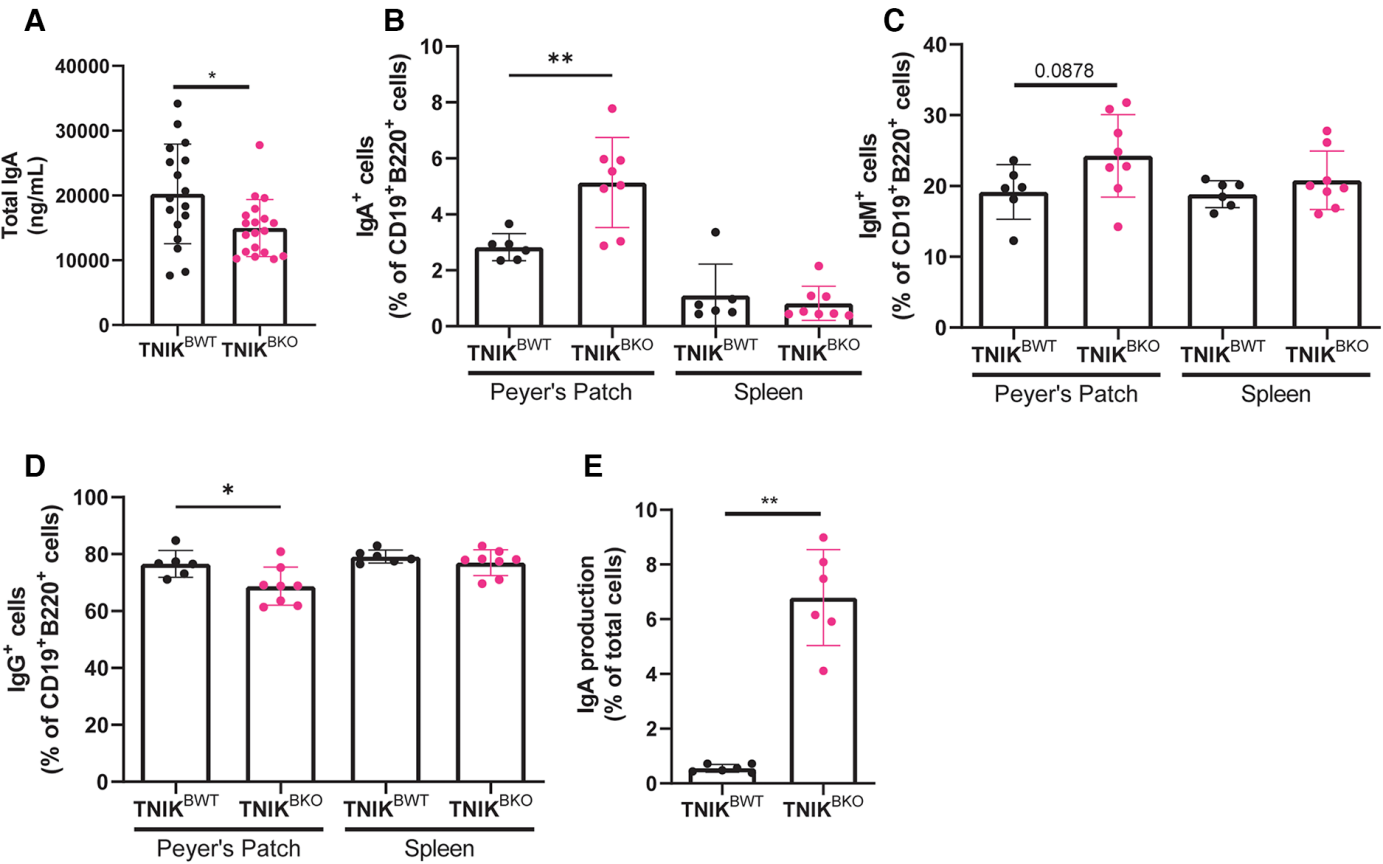
TNIK B cell deficiency increases IgA production. (**A**) Blood plasma was isolated and analyzed for IgA levels, which were decreased in *TNIK^BKO^* compared to *TNIK^BWT^* mice (*n* = 17/19). (**B–D**) Cells from intestinal Peyer's Patches and spleen were analyzed via flow cytometry for IgA^+^, IgM^+^ and IgG^+^ expressing cells (CD45^+^) (*n* = 6/8). (**E**) Splenic resting B cells (CD43^−^) were isolated and stimulated with αCD40, IL-4, IL-5, TGF-β and αIgD to differentiate into plasma cells, which were analyzed via flow cytometry for IgA expression (*n* = 6 per genotype).

Interestingly, *TNIK^BKO^* mice displayed a reduction in total plasma IgA levels (Figure 4A). IgA is an antibody-subtype involved in mucosal homeostasis, and is produced mainly in the gut. The intestinal Peyer's patches, small lymphoid aggregates, are known to be the main producers of gut IgA (26). However, in contrast to the observed reduction of plasma IgA, Peyer's patches in *TNIK^BKO^* mice contained an increased number of IgA^+^ B cells (Figure 4B). The numbers of IgM^+^ and IgG^+^ B cells did not differ (Figures 4C–D). This is in contrast to the spleen, where the number of IgA producing B cells was unchanged, suggesting a gut specific effect in the induction of IgA production in the intestine, but not spleen. In Peyer's patches, B cells isotype switch to IgA under the influence of transforming growth factor β (TGF-β) (27). And indeed, treatment of naive B cells isolated from *TNIK^BKO^*mice showed an increase isotype switching compared to B cells from *TNIK^BWT^* mice. (Figure 4E). These data suggest that TNIK deficient B cells in the gut exhibit enhanced TGF-β mediated IgA isotype switching. However, this TNIK-deficiency induced increase in mucosal IgA did not affect atherosclerosis in our model.

### 3.4. B cell specific TNIK deletion does not affect other immune cell types or subsets

**Figure 5:**
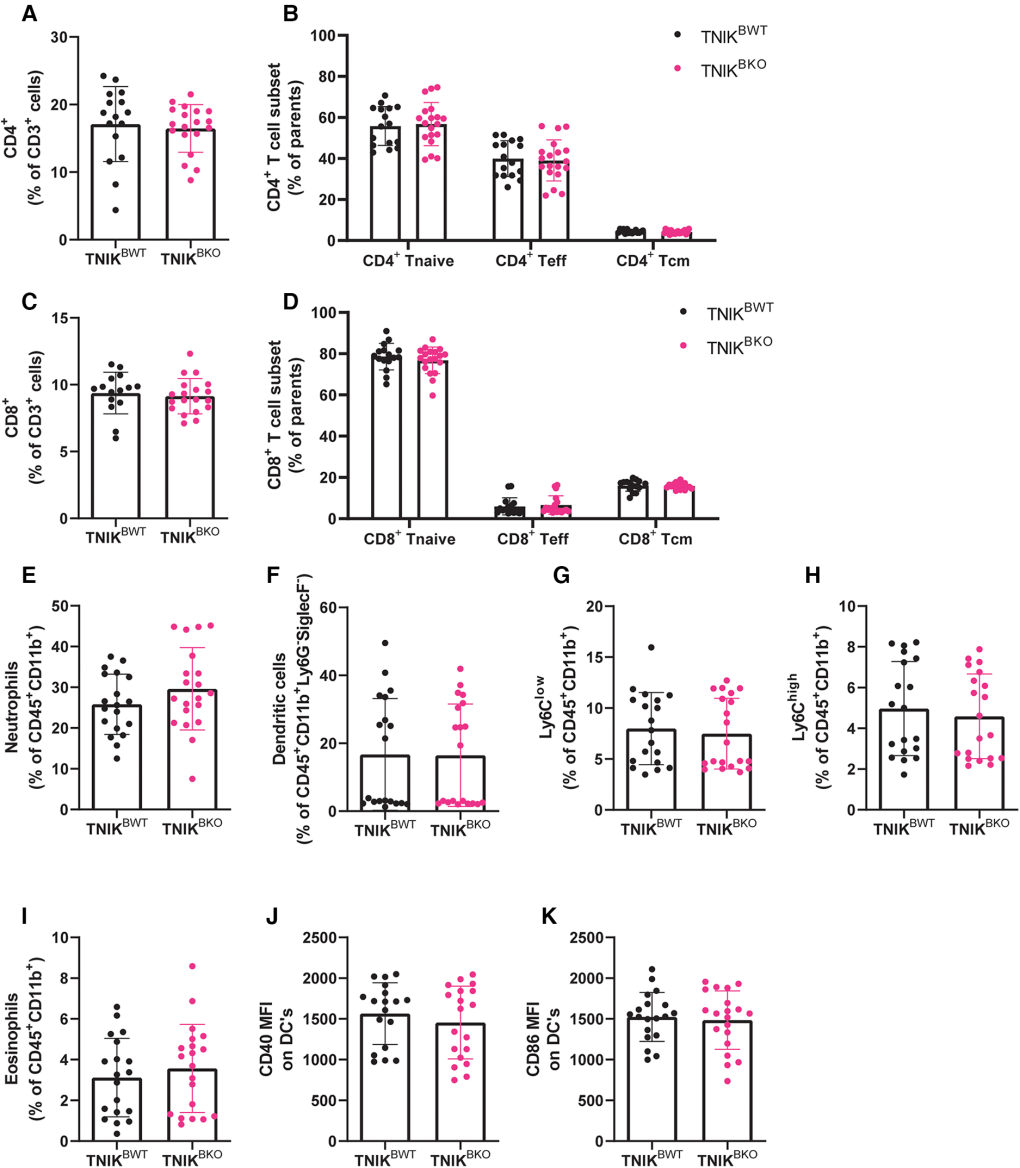
B cell TNIK deficiency does not affect T cell ratios or myeloid populations in the spleen. The CD4^+^ and CD8^+^ T cells from the spleens of *TNIK^BWT^* and *TNIK^BKO^* mice were analyzed via flow cytometry. (**A**) Total CD4^+^ T cells, alongside (**B**) their subsets, naive (CD44^−^CD62L^+^), effector (CD44^+/−^CD62L^−^) and central memory T cells (CD44^+^ CD62L^+^), are unaffected by B cell TNIK deficiency (*n* = 16/20). (**C**) Total CD8^+^ T cells and (**D**) its subsets, naive (CD44^−^CD62L^+^), effector (CD44^+/−^CD62L^−^) and central memory (CD44^+^ CD62L^+^), are unaltered in the *TNIK^BKO^* compared to *TNIK^BWT^* mice (*n* = 15/19). Splenic myeloid cells (CD45^+^ CD11b^+^) were also analyzed by flow cytometry. (**E**) Neutrophils (ly6G^+^SiglecF^−^), (**F**) dendritic cells (MHCII^+^CD11c^+^), (**G**) non-classical Ly6C^low^ monocytes, (**H**) classical Ly6C^high^ monocytes and (**I**) eosinophils (Ly6G^low^SiglecF^+^) were unaffected by genotype. Further activation status of dendritic cells by measuring (**J**) CD40 and (**K**) CD86 expression revealed no differences between *TNIK^BWT^* and *TNIK^BKO^* mice [(**E,G–K**) *n* = 19/20, and (**F**) *n* = 10/10].

*TNIK^BKO^* mice displayed similar amounts of CD4^+^ T cells, CD8^+^ T cells, naive, effector and central memory CD4^+^ and CD8^+^ T cells in blood, spleen and lymph nodes (Figures 5A–D; Supplementary Figure 5). Furthermore, the myeloid compartment, containing neutrophils, dendritic cells, Ly6C^low^ and Ly6C^high^ monocytes, and eosinophils, were not affected in spleen and blood by TNIK deficiency in B cells (Figures 5E–K; Supplementary Figure S6). In addition, stem cells and mature immune cells present in the bone marrow were unaffected by B-cell deficiency of TNIK (Supplementary Figure S7).

## 4. Discussion

In the current paper, we investigated the role of B cell specific TRAF2- and NCK-interacting kinase (TNIK) in atherosclerosis. We conclude that TNIK signaling in B cells does not affect atherogenesis, nor did it affect immunological pathways relevant for atherosclerosis.

There is limited research on the role of TNIK in B cells (11). TNIK has been shown to be a crucial partner in the signaling pathway of latent membrane protein 1 (LMP1), an Epstein-Barr virus (EBV) oncogene with many similarities to CD40 (28). Here TNIK was shown to bind TRAF6 and TRAF2 (11). Thereby activating the nuclear factor kappa B (NF-κB) signaling pathway inducing proliferation and activation of B cells (29). Furthermore, the authors suggest that TNIK activation via LMP1 might not induce Wnt signaling. However, in our *TNIK^BKO^* mice, no reduction in NF-κB phosphorylation was measured after activating CD40 signaling in B cells, despite a 90% reduction in B-cell specific TNIK expression. One limitation of the study was the use of HEK293 cells as a model for CD40 and LMP1 signaling (11). This suggests that while TNIK may be associated with TRAF6 when co-transfected with LMP1 in HEK293 cells, this pathway may not be functional in primary B cells. Furthermore, these outcomes highlight functional differences between LMP1 and CD40. While it was shown that LMP1 and CD40 have many similarities in their signaling pathways, LMP1's intracellular signaling domain differs from CD40 (30). Even though both mainly signal via TRAFs, they bind these with different affinities. Furthermore, LMP1 but not CD40 engages tumor necrosis factor receptor-associated death domain (TRADD) for its signaling (31). While CD40 signaling promotes germinal center formation, LMP1 inhibits this, instead promoting extrafollicular B cell activation (30). Another key difference between CD40 and LMP1 is the dependence on (TNF)-receptor 1–associated death domain protein (TRADD) for its TRAF6 signaling. While CD40 can directly bind TRAF6, LMP1 cannot (28, 32), and is dependent on TRADD (33, 34). Further, LMP1 and CD40 downstream signaling differs with regard to the functions of the kinases TAK1, IKK2 and TPL2 (35). This is contrasted by CD40, which does not bind TRADD. This suggest that results found in EBV transformed B cells expressing LMP1 are not directly translatable to CD40 signaling in primary B cells.

Interestingly, when we analyzed atherosclerosis in B cell TNIK deficient atherosclerotic mice, we did not observe any effects on plaque size or composition, with the exception of a trend towards a decrease in plaque macrophage content. We have no solid explanation for this, but the increase of intestinal IgA may have altered the microbiome and it can be speculated that this may have shifted the immune system towards a slightly more immunomodulatory state, thereby reducing leukocyte recruitment. Furthermore, no B cell subsets differences were detected, nor any indirect effects on other cell types. This is unexpected, as TNIK plays a role key role in Wnt signaling, a pathway with a role in several immune related diseases such as Rheumatoid Arthritis and Ankylosing Spondylitis (36). Moreover, this pathways has a known role in atherogenesis (37). In acute lymphocytic choriomeningitis virus (LCMV), deletion of TNIK on CD8^+^ T cells via the UBC-Cre tamoxifen system impaired the formation of memory cells due to defective signaling in the Wnt pathway (17). Furthermore, TNIK deficient effector CD8^+^ T cells displayed increased proliferation, apoptosis and reprogramming towards a higher glycolytic metabolic profile (17). In colorectal cancer, over 90% of tumors exhibit mutations in the Wnt/β-catenin signaling pathway. TNIK was identified as a promising target to inhibit Wnt-signaling due to its binding to TCF-4/β-catenin complex (15). In colorectal cancer, it was found that TNIK is necessary for tumor-initiating function of stem cells in the intestines, and in acute myeloid leukemia blasts TNIK reduces survival rate due to the activation of Wnt signaling (38, 39). As B cells are regulated by the Wnt signaling pathway to induce proliferation (40), we anticipated similar effects within our B cell specific mouse model. However, we could not identify any differences in B cell differentiation and subset distribution. Furthermore, no secondary changes were observed in T cell or myeloid population in our atherosclerotic model, indicating that B cell TNIK deficiency does not indirectly affect other immune cells.

Interestingly, we found a strong increase in IgA producing B cells in the small intestine's Peyer's patches. Something which is in contrast to the observed decrease in blood IgA while no difference in splenic IgA producing B cells was observed. While significant redundancy exists, *in vivo* isotype switching to IgA is mostly controlled by transforming growth factor-β (TGF-β) (41, 42). One key difference between the splenic environment and that on the Payer's patches is the local production of TGF-β by stimulation of immune cells by the microbiome (43). The potential role of TNIK on TGF-β signaling was highlighted by *in vitro* experiments where naïve splenic B cells were isolated and found to more efficiently isotype switch to IgA production under the influence of TGF-β. It has been shown that the kinase family where TNIK belongs to can interact with, and inhibit the binding of Smad proteins to the TGF-β receptor, thereby inhibiting TGF-β signaling (44).

There are some limitations to our current experimental setup. First, we only used female mice, who develop atherosclerosis faster than their male counterparts, and of course, have different estrogen levels than male mice. Second, we used mice that were fed a high cholesterol diet, and did not do a normal chow diet group, which would have developed atherosclerosis more slowly. We have only investigated relatively advanced atheroma's and did not elucidate the role of B cell TNIK deficiency on early plaque formation, yet we do feel that the effects of B cell TNIK on atherogenesis are minor.

Although our data do not show any effect of B cell TNIK deficiency on atherosclerosis, B cell TNIK may play an important role in IgA mediated diseases. The majority of IgA is produced by B cells in Peyer's patches (45). The fact that absence of TNIK in B cells increases IgA production in Peyer's patches suggests that inhibition of TNIK could increase microbiota diversity and metabolism of gut microbiota (46). Increases in intestinal IgA production are correlated with IgA-mediated diseases, including IgA nephropathies (47) and inflammatory bowel diseases (48). Activating B cell TNIK could be a potential target to lower IgA levels and ameliorate IgA-associated diseases.

In conclusion, TNIK signaling in B cells does not play a role in the pathogenesis of atherosclerosis. However, our data suggest that B-cell TNIK signaling may be relevant for inhibiting TGF-β mediated IgA switching of B cells.

## Data Availability

The raw data supporting the conclusions of this article will be made available by the authors, without undue reservation.

## Supplementary material

Supplementary Figure 1 B cell specific TNIK deficiency. TNIK deficiency of *ApoE^−/−^TNIK^fl/fl^* (*TNIK^BWT^*) and *ApoE^−/−^TNIK^fl/fl^CD19-cre* (*TNIK^BKO^*) mice was validated in isolated splenic B cells (*n* = 2 or 3/genotype of 4 individual experiments).

Supplementary Figure 2 B cell TNIK deficiency does not affect aortic root plaque size. Aortic root of TNIK^BWT^ and TNIK^BKO^ mice were isolated and examined for the atherosclerotic plaque size. Area under the curve of the plaque volume throughout the atherosclerotic plaque formation did not differ between genotypes (*n* = 19/20).

Supplementary Figure 3 Blood B cell differentiation and subsets unaffected in *TNIK^BKO^* compared to *TNIK^BWT^* mice. The differentiation and subsets of B cells are measured by flow cytometry in blood from *TNIK^BWT^* and *TNIK^BKO^* mice. (**A**) The total number of leukocytes (CD45^+^) per µl blood was unaffected between *TNIK^BWT^* and *TNIK^BKO^* mice (*n* = 9/10). (**B**) Transitional stage T-1 (CD19^+^B220^+^IgM^+^CD23^−^) (*n* = 19/19) and (**C**) T-2 (CD19^+^B220^+^IgM^+^CD23^−^) (*n* = 19/20) are unaffected in *TNIK^BKO^* compared to *TNIK^BWT^* mice. The (**D**) number of B1 cell per μl blood (CD19^low^B220^+^) (*n* = 9/8), (**E**) fraction of B1 cells (*n* = 17/19), (**F**) number of B2 cells (CD19^+^B220^+^) per μl blood, (*n* = 9/10), (**G**) fraction of B2 cell (*n* = 19/20) are similar between the genotypes, as are the (**H**) marginal zone (CD19^+^B220^+^CD21^+^CD23^−^) (*n* = 19/20) and (**I**) follicular B cells (CD19^+^B220^+^CD21^low^CD23^+^) (*n* = 19/20).

Supplementary Figure 4 B cell specific TNIK deficiency does not affect canonical NF-κB pathway by activation of CD40. (**A**) Expression of the p65 subunit from NF-κB expression the (**B**) phosphorylated P65 expression was measured in *TNIK^BWT^* and *TNIK^BKO^* isolated splenic B cells (*n* = 5/6). (**C**) Ratio of phosphorylated NF-κB over NF-κB measured in *TNIK^BWT^* and *TNIK^BKO^* isolated splenic B cells.

Supplementary Figure 5 T cell subsets from lymph nodes and blood are not altered due to B cell TNIK deficiency. (**A**) Fraction of CD4^+^ T cells in lymph nodes (gated from total cells) (*n* = 19/20) and (**B**) CD4^+^ naive (CD44^−^CD62L^+^), effector (CD44^+/−^CD62L^−^) and central memory T cells (CD44^+^CD62L^+^) (*n* = 19/20), are unaffected by B cell TNIK deficiency (*n* = 16/20). (**C**) Fraction of CD8^+^ T cells in lymph nodes (gated from total cells) (*n* = 19/20) and (**D**) CD8^+^ naive (CD44^−^CD62L^+^), effector (CD44^+/−^CD62L^−^) and central memory T cells (CD44^+^CD62L^+^) (*n* = 19/20), are unaffected by B cell TNIK deficiency (*n* = 16/20). (**E**) Fraction of CD4^+^ T cells in blood (gated from total cells) (*n* = 19/20) and (**F**) CD4^+^ naive (CD44^−^CD62L^+^), effector (CD44^+/−^CD62L^−^) and central memory T cells (CD44^+^CD62L^+^) (*n* = 19/20), are unaffected by B cell TNIK deficiency (*n* = 16/20). (**G**) Fraction of CD8^+^ T cells in blood (gated from total cells) (*n* = 19/20) and (**H**) CD8^+^ naive (CD44^−^CD62L^+^), effector (CD44^+/−^CD62L^−^) and central memory T cells (CD44^+^CD62L^+^) (*n* = 19/20), are unaffected by B cell TNIK deficiency (*n* = 16/20).

Supplementary Figure 6 Myeloid immune cells in the blood largely unaffected by TNIK B cell deficiency. Blood myeloid (CD45^+^CD11b^+^) cells were analyzed by flow cytometry from *TNIK^BWT^* and *TNIK^BKO^* mice. (**A**) Neutrophils (ly6G^+^SiglecF^−^) are unaffected in *TNIK^BKO^* compared to *TNIK^BWT^* mice (*n* = 9/10). (**B**) Eosinophils (Ly6G^low^SiglecF^+^) (*n* = 9/10), (**C**) non-classical Ly6C^low^ monocytes (*n* = 8/10), (**D**) classical Ly6C^high^ monocytes (*n* = 9/10), (**E**) dendritic cells (MHCII^+^CD11c^+^), (*n* = 9/10) are unaffected by genotype. Further activation status of dendritic cells by measuring (**G**) CD40 and (**H**) CD86 expression revealed no differences between *TNIK^BWT^* and *TNIK^BKO^* mice (*n* = 9/10).

Supplementary Figure 7 Bone marrow differentiation unaffected due to B cell TNIK deficiency. Flow cytometric identification of bone marrow hematopoietic stem cells from *TNIK^BWT^* and *TNIK^BKO^* mice. (**A**) Total number of hematopoietic stem cells (Lineage^−^Sca-1^+^c-Kit^+^) showed a trend towards a decrease in *TNIK^BKO^* mice (*n* = 19/20). (**B**) Characterization of total number of long term stem cell (LT-SC: Lineage^−^Sca-1^+^c-Kit^+^CD150^+^CD48^−^), short term stem cell (ST-SC: Lineage^−^Sca-1^+^c-Kit^+^CD150^−^CD48^−^) showed no differences between genotypes (*n* = 19/20). Furthermore, no difference in multipotent progenitor cells (MPP: Sca-1^+^c-Kit^+^) or MPP subpopulations (MPP1a: CD48^−^CD150^−^135^+^, MPP1b: CD48^−^CD150^−^135^+^, MPP2: CD48^+^CD150^+^, MPP3:CD48^+^CD150^−^CD135^+^, MPP4:CD48^+^CD150^−^CD135^+^) was observed (*n* = 19/20). (**C**) Common lymphoid progenitors (CLP: Lineage^−^Sca-1^low^c-Kit^+^CD48^+^CD16/CD32^−^) – early common lymphoid progenitor (CD127^+^CD135^+^), CLP (CD127^+^CD135^−^CD27^−^) and late CLP (CD127^+^CD135^−^CD27^+^) – did not reveal differences between *TNIK^BWT^* and *TNIK^BKO^* mice (*n* = 19/20). (**D**) Myeloid progenitors (Sca-1^−^cKit^+^) – granulocyte-monocyte progenitor (GMP: (CD16/32^+^), common myeloid progenitor (CMP: CD16/32^low^) and megakaryocyte-erythrocyte progenitor (MEP: CD16/32^−^) – also do not differ between *TNIK^BWT^* and *TNIK^BKO^* mice (*n* = 19/20). (**E**) Number of mature immune (CD45^+^) cells, Plasma B cells (CD138^+^), CD4+ and CD8+ T cells (CD3^+^) in the bone marrow are not affected by TNIK B cell deficiency (*n* = 9/10).

Supplementary Figure 8 Bone marrow differentiation unaffected due to B cell TNIK deficiency. Flow cytometric identification of bone marrow hematopoietic stem cells from *TNIK^BWT^* and *TNIK^BKO^* mice. (**A**) Total number of hematopoietic stem cells (Lineage^−^Sca-1^+^c-Kit^+^) showed a trend towards a decrease in *TNIK^BKO^* mice (*n* = 19/20). (**B**) Characterization of total number of long term stem cell (LT-SC: Lineage^−^Sca-1^+^c-Kit^+^CD150^+^CD48^−^), short term stem cell (ST-SC: Lineage^−^Sca-1^+^c-Kit^+^CD150^−^CD48^−^) showed no differences between genotypes (*n* = 19/20). Furthermore, no difference in multipotent progenitor cells (MPP: Sca-1^+^c-Kit^+^) or MPP subpopulations (MPP1a: CD48^−^CD150^−^135^+^, MPP1b: CD48^−^CD150^−^135^+^, MPP2: CD48^+^CD150^+^, MPP3:CD48^+^CD150^−^CD135^+^, MPP4:CD48^+^CD150^−^CD135^+^) was observed (*n* = 19/20). (**C**) Common lymphoid progenitors (CLP: Lineage^−^Sca-1^low^c-Kit^+^CD48^+^CD16/CD32^−^) – early common lymphoid progenitor (CD127^+^CD135^+^), CLP (CD127^+^CD135^−^CD27^−^) and late CLP (CD127^+^CD135^−^CD27^+^) – did not reveal differences between *TNIK^BWT^* and *TNIK^BKO^* mice (*n* = 19/20). (**D**) Myeloid progenitors (Sca-1^−^cKit^+^) – granulocyte-monocyte progenitor (GMP: (CD16/32^+^), common myeloid progenitor (CMP: CD16/32^low^) and megakaryocyte-erythrocyte progenitor (MEP: CD16/32^−^) – also do not differ between *TNIK^BWT^* and *TNIK^BKO^* mice (*n* = 19/20). (**E**) Number of mature immune (CD45^+^) cells, Plasma B cells (CD138^+^), CD4+ and CD8+ T cells (CD3^+^) in the bone marrow are not affected by TNIK B cell deficiency (*n* = 9/10).

Supplementary Method 1 Gating strategy for flow cytometry on spleen, LN and blood samples. First three gating steps seen in figure (**A**) are applied to all other gating strategies. (**A**) Gating strategy for germinal center plasma cells. (**B**) Gating strategy for B cell differentiation subsets. (**C**) Gating strategy for CD4+ and CD8+ T cell subsets. (**D**) Gating strategy for myeloid cell populations.

Supplementary Method 2 Gating strategy for flow cytometry on Peyer’s patches and spleen. (**A**) Gating strategy for IgA+ cells in Peyer’s Patches and spleen. (**B**) Gating strategy for IgM+ and IgG+ plasma cells cultured in vitro.

Supplementary Method 3 Gating strategy for stem cells. Gating strategy for stem cells from bone marrow samples.
