# Supplementary figures and images for: Deficiency of germinal center kinase TRAF2 and NCK-interacting kinase (TNIK) in B cells does not affect atherosclerosis
Source: Front Cardiovasc Med. 2023 May 5;10:1171764. doi: 10.3389/fcvm.2023.1171764 (PMC10196212; doi:10.3389/fcvm.2023.1171764)

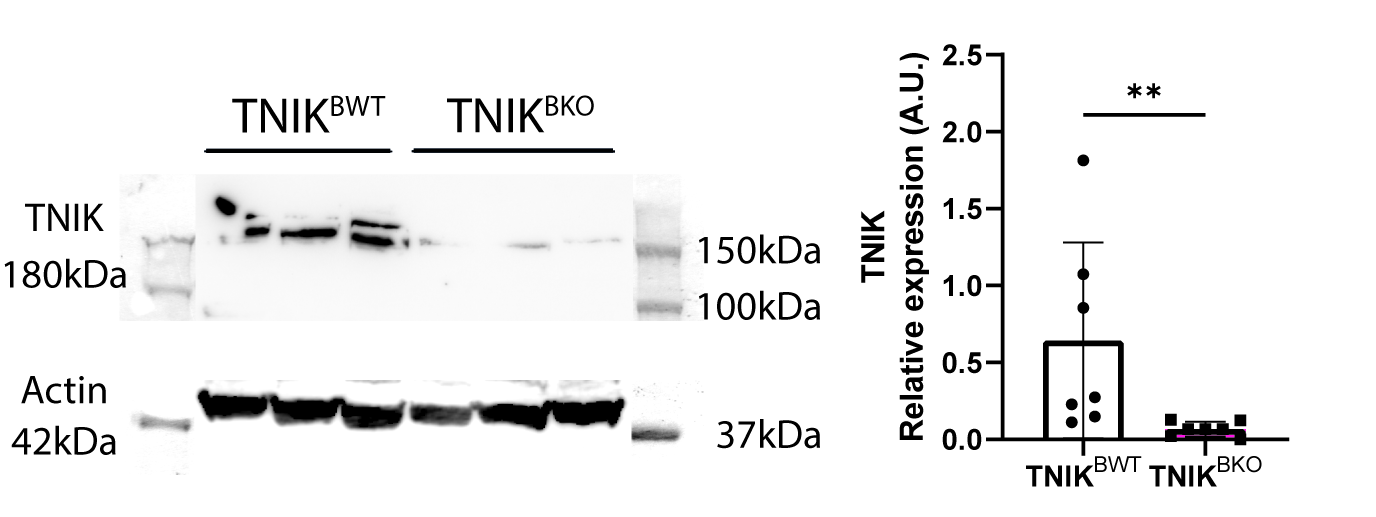

Supplement: Supplementary Figure 1 — B cell specific TNIK deficiency. TNIK deficiency of ApoE−/−TNIKfl/fl (TNIKBWT) and ApoE−/−TNIKfl/flCD19-cre (TNIKBKO) mice was validated in isolated splenic B cells (n = 2 or 3/genotype of 4 individual experiments). [file Image1.tif]

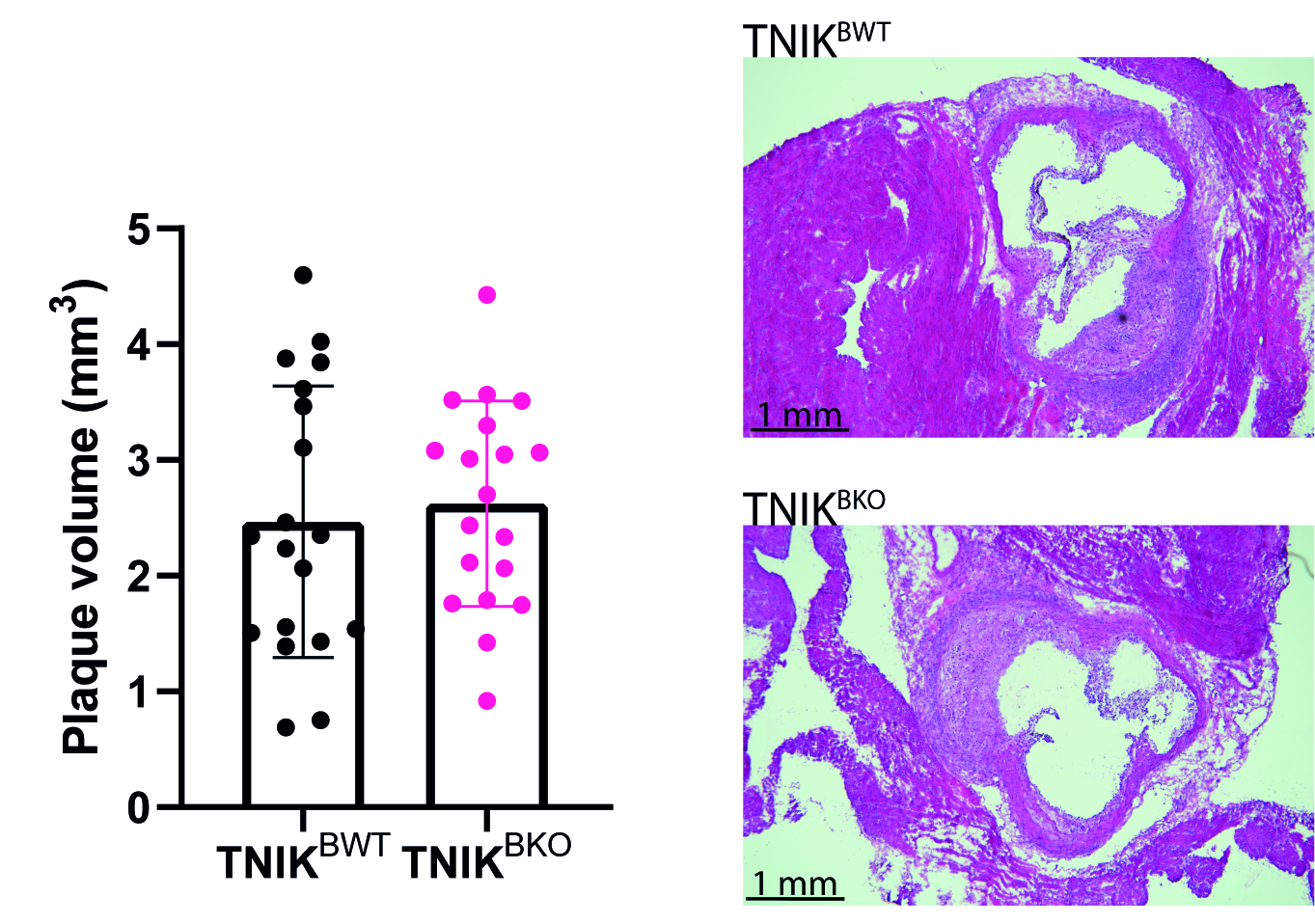

Supplement: Supplementary Figure 2 — B cell TNIK deficiency does not affect aortic root plaque size. Aortic root of TNIKBWT and TNIKBKO mice were isolated and examined for the atherosclerotic plaque size. Area under the curve of the plaque volume throughout the atherosclerotic plaque formation did not differ between genotypes (n = 19/20). [file Image2.tif]

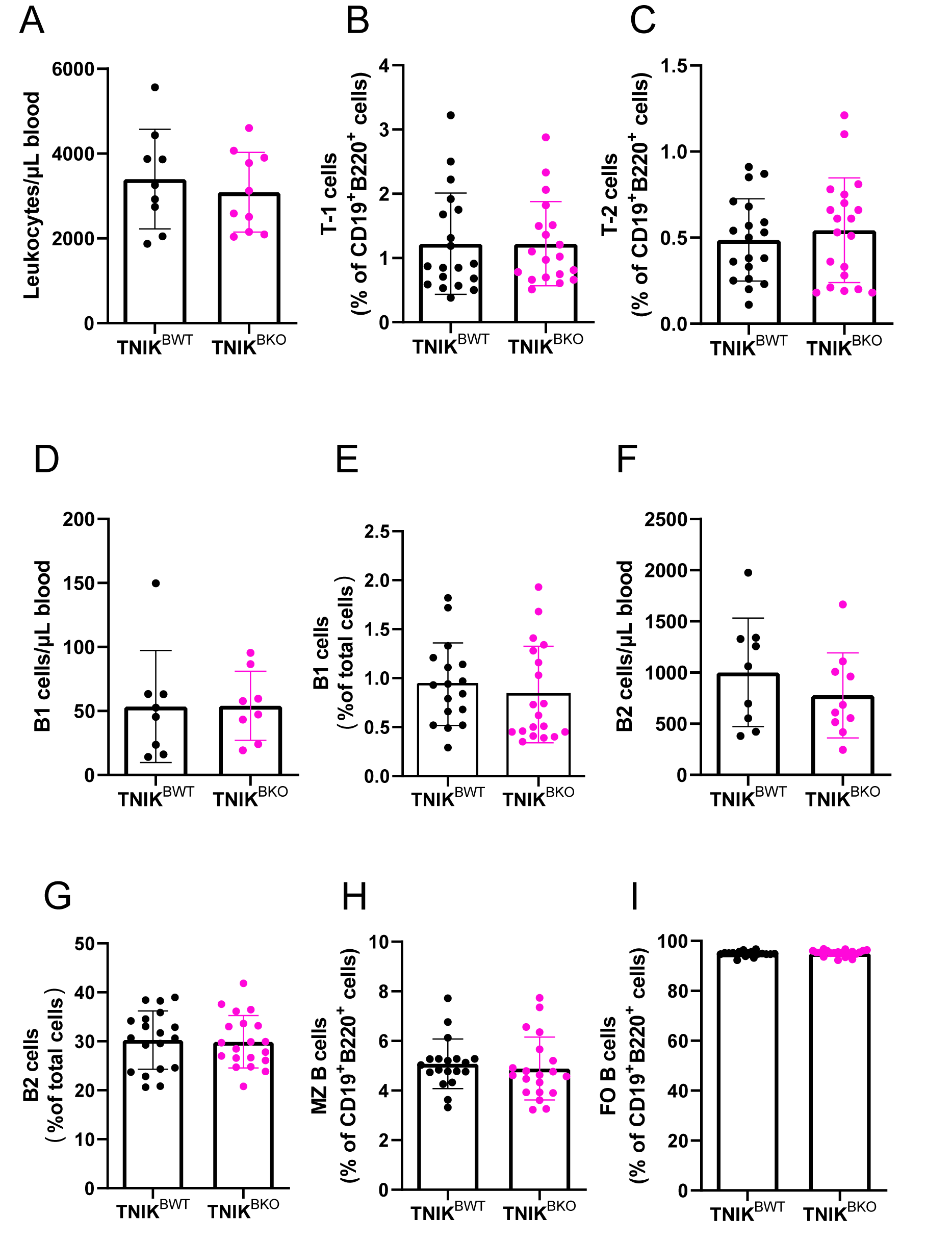

Supplement: Supplementary Figure 3 — Blood B cell differentiation and subsets unaffected in TNIKBKO compared to TNIKBWT mice. The differentiation and subsets of B cells are measured by flow cytometry in blood from TNIKBWT and TNIKBKO mice. (A) The total number of leukocytes (CD45+) per µl blood was unaffected between TNIKBWT and TNIKBKO mice (n = 9/10). (B) Transitional stage T-1 (CD19+B220+IgM+CD23−) (n = 19/19) and (C) T-2 (CD19+B220+IgM+CD23−) (n = 19/20) are unaffected in TNIKBKO compared to TNIKBWT mice. The (D) number of B1 cell per μl blood (CD19lowB220+) (n = 9/8), (E) fraction of B1 cells (n = 17/19), (F) number of B2 cells (CD19+B220+) per μl blood, (n = 9/10), (G) fraction of B2 cell (n = 19/20) are similar between the genotypes, as are the (H) marginal zone (CD19+B220+CD21+CD23−) (n = 19/20) and (I) follicular B cells (CD19+B220+CD21lowCD23+) (n = 19/20). [file Image3.tif]

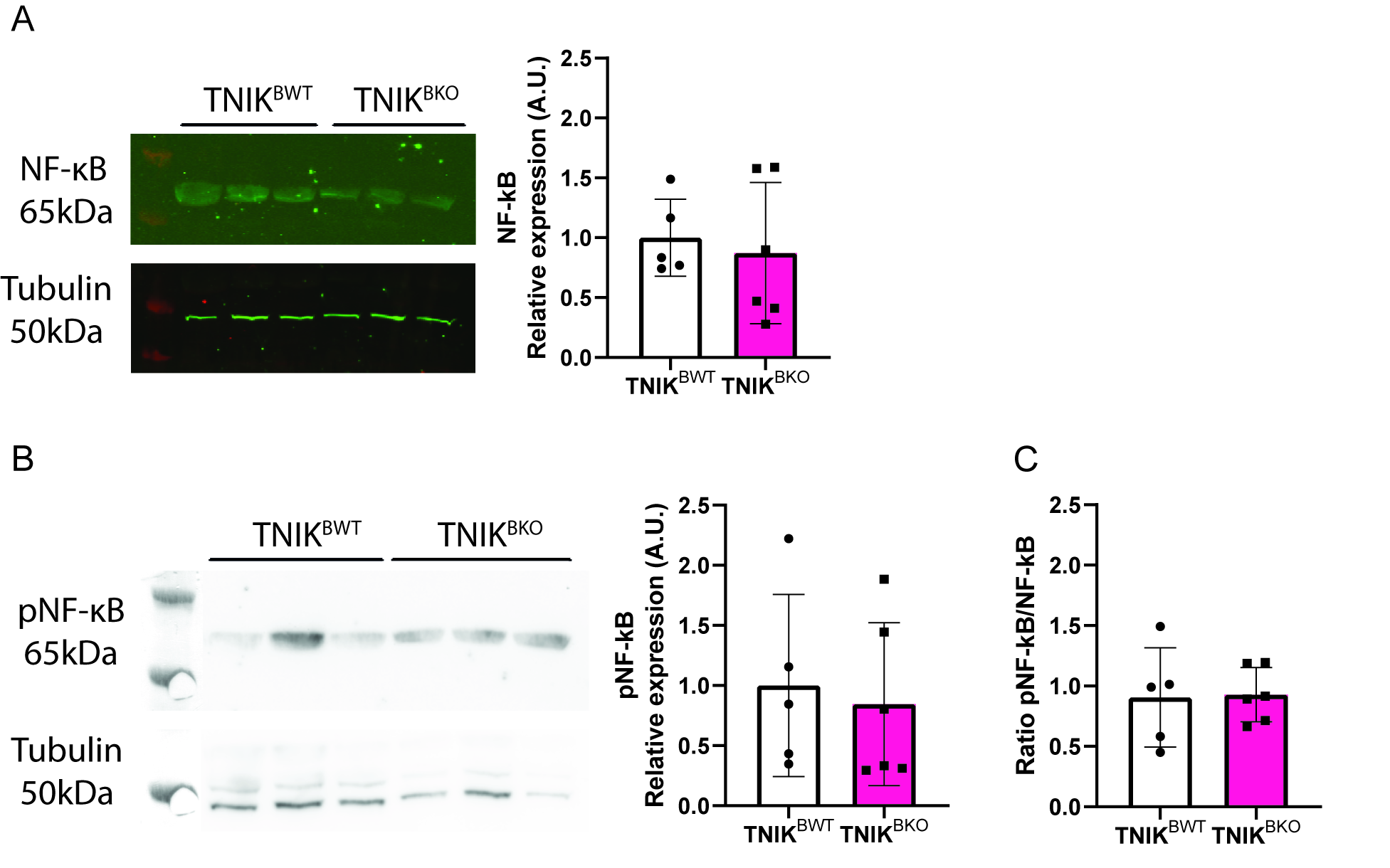

Supplement: Supplementary Figure 4 — B cell specific TNIK deficiency does not affect canonical NF-κB pathway by activation of CD40. (A) Expression of the p65 subunit from NF-κB expression the (B) phosphorylated P65 expression was measured in TNIKBWT and TNIKBKO isolated splenic B cells (n = 5/6). (C) Ratio of phosphorylated NF-κB over NF-κB measured in TNIKBWT and TNIKBKO isolated splenic B cells. [file Image4.tif]

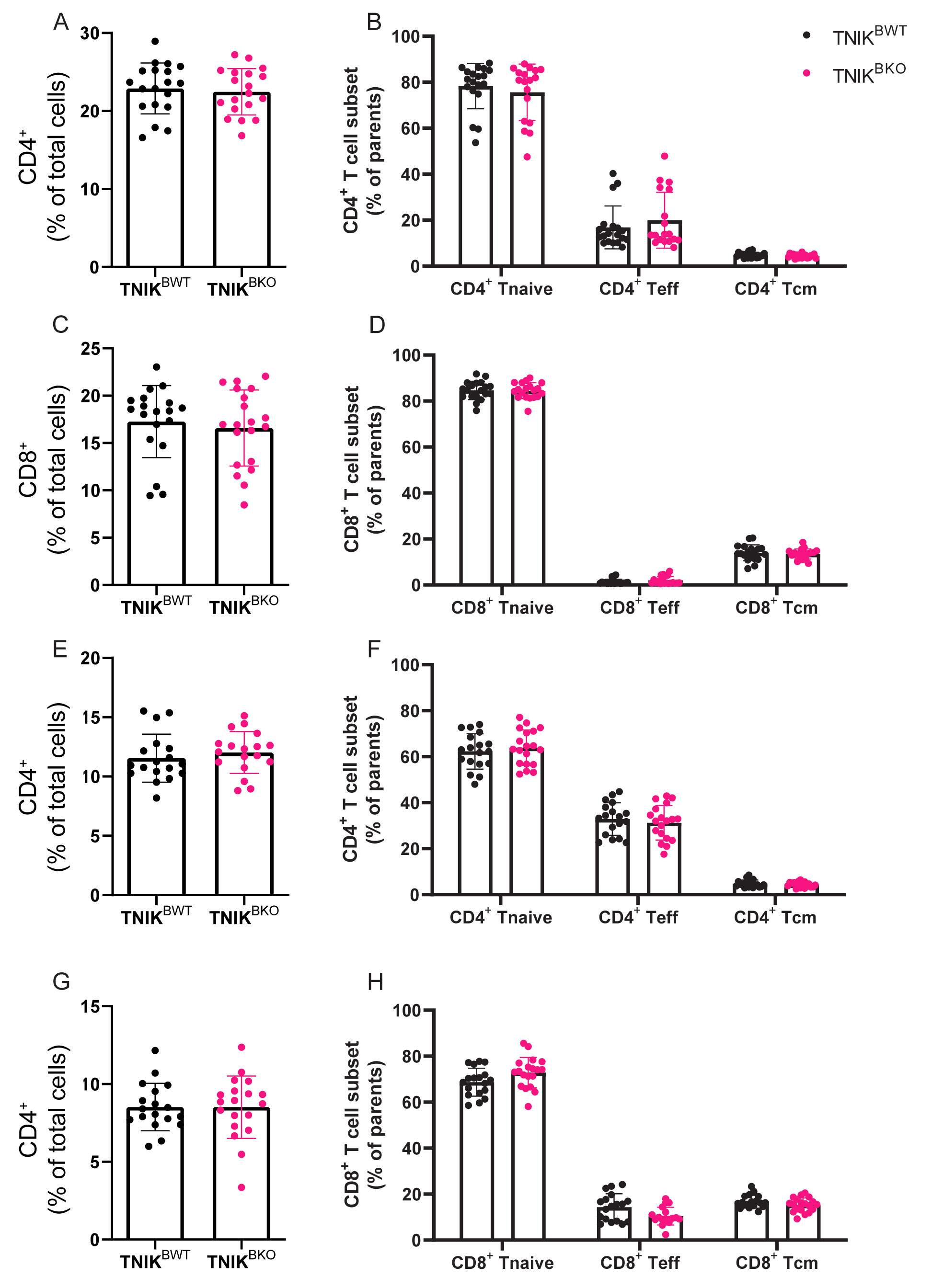

Supplement: Supplementary Figure 5 — T cell subsets from lymph nodes and blood are not altered due to B cell TNIK deficiency. (A) Fraction of CD4+ T cells in lymph nodes (gated from total cells) (n = 19/20) and (B) CD4+ naive (CD44−CD62L+), effector (CD44+/−CD62L−) and central memory T cells (CD44+CD62L+) (n = 19/20), are unaffected by B cell TNIK deficiency (n = 16/20). (C) Fraction of CD8+ T cells in lymph nodes (gated from total cells) (n = 19/20) and (D) CD8+ naive (CD44−CD62L+), effector (CD44+/−CD62L−) and central memory T cells (CD44+CD62L+) (n = 19/20), are unaffected by B cell TNIK deficiency (n = 16/20). (E) Fraction of CD4+ T cells in blood (gated from total cells) (n = 19/20) and (F) CD4+ naive (CD44−CD62L+), effector (CD44+/−CD62L−) and central memory T cells (CD44+CD62L+) (n = 19/20), are unaffected by B cell TNIK deficiency (n = 16/20). (G) Fraction of CD8+ T cells in blood (gated from total cells) (n = 19/20) and (H) CD8+ naive (CD44−CD62L+), effector (CD44+/−CD62L−) and central memory T cells (CD44+CD62L+) (n = 19/20), are unaffected by B cell TNIK deficiency (n = 16/20). [file Image5.tif]

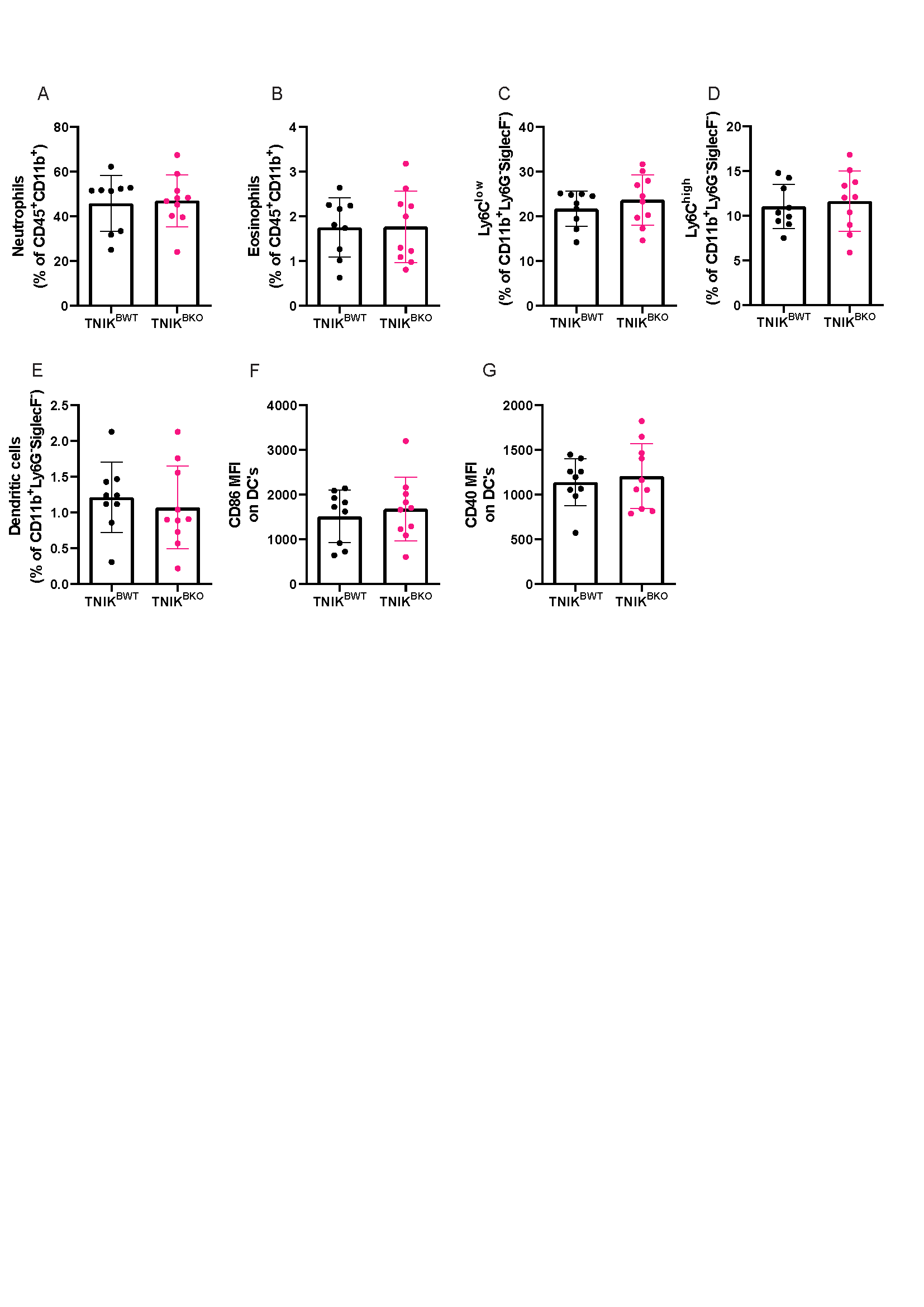

Supplement: Supplementary Figure 6 — Myeloid immune cells in the blood largely unaffected by TNIK B cell deficiency. Blood myeloid (CD45+CD11b+) cells were analyzed by flow cytometry from TNIKBWT and TNIKBKO mice. (A) Neutrophils (ly6G+SiglecF−) are unaffected in TNIKBKO compared to TNIKBWT mice (n = 9/10). (B) Eosinophils (Ly6GlowSiglecF+) (n = 9/10), (C) non-classical Ly6Clow monocytes (n = 8/10), (D) classical Ly6Chigh monocytes (n = 9/10), (E) dendritic cells (MHCII+CD11c+), (n = 9/10) are unaffected by genotype. Further activation status of dendritic cells by measuring (G) CD40 and (H) CD86 expression revealed no differences between TNIKBWT and TNIKBKO mice (n = 9/10). [file Image6.tiff]

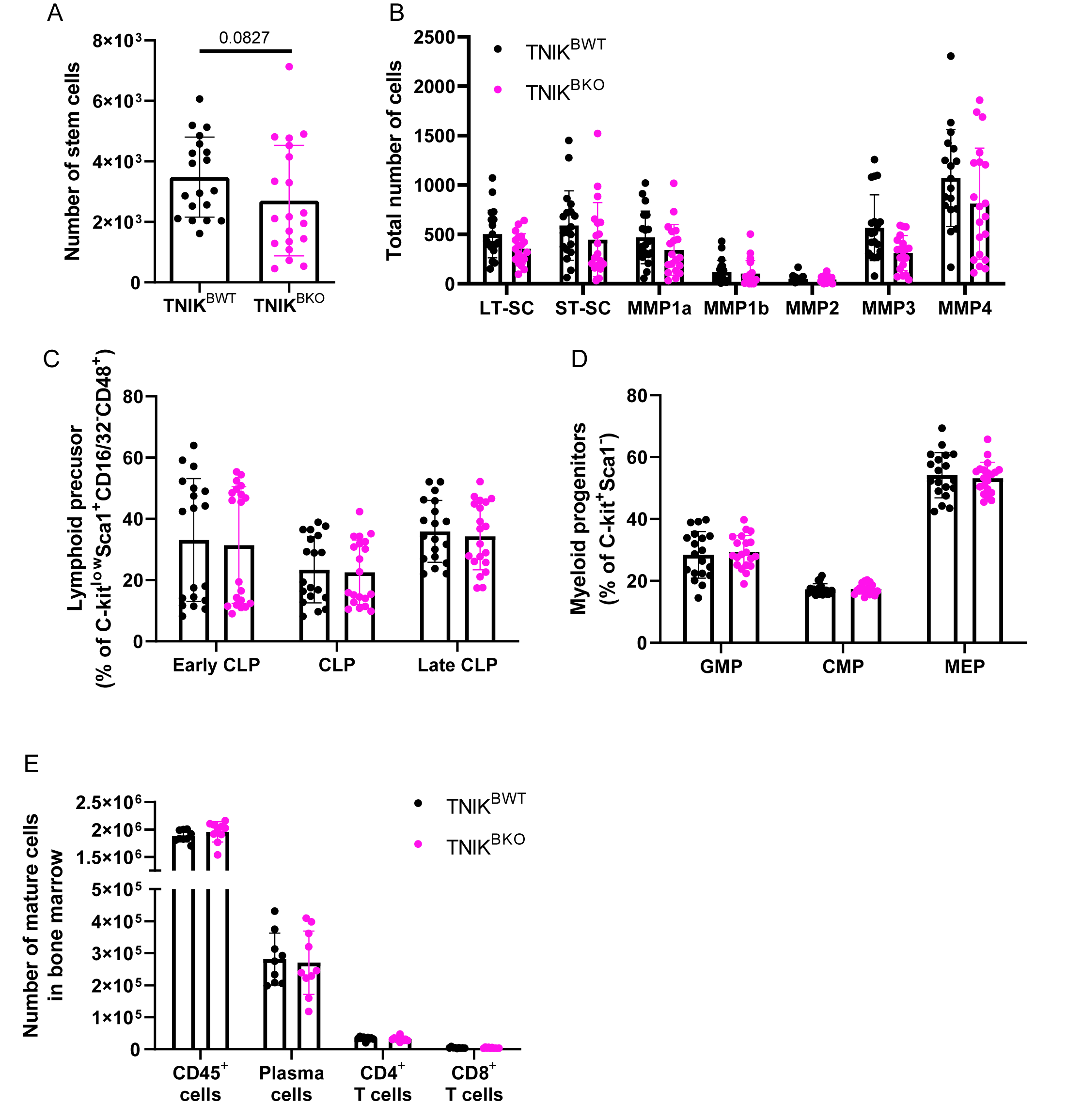

Supplement: Supplementary Figure 7 — Bone marrow differentiation unaffected due to B cell TNIK deficiency. Flow cytometric identification of bone marrow hematopoietic stem cells from TNIKBWT and TNIKBKO mice. (A) Total number of hematopoietic stem cells (Lineage−Sca-1+c-Kit+) showed a trend towards a decrease in TNIKBKO mice (n = 19/20). (B) Characterization of total number of long term stem cell (LT-SC: Lineage−Sca-1+c-Kit+CD150+CD48−), short term stem cell (ST-SC: Lineage−Sca-1+c-Kit+CD150−CD48−) showed no differences between genotypes (n = 19/20). Furthermore, no difference in multipotent progenitor cells (MPP: Sca-1+c-Kit+) or MPP subpopulations (MPP1a: CD48−CD150−135+, MPP1b: CD48−CD150−135+, MPP2: CD48+CD150+, MPP3:CD48+CD150−CD135+, MPP4:CD48+CD150−CD135+) was observed (n = 19/20). (C) Common lymphoid progenitors (CLP: Lineage−Sca-1lowc-Kit+CD48+CD16/CD32−) – early common lymphoid progenitor (CD127+CD135+), CLP (CD127+CD135−CD27−) and late CLP (CD127+CD135−CD27+) – did not reveal differences between TNIKBWT and TNIKBKO mice (n = 19/20). (D) Myeloid progenitors (Sca-1−cKit+) – granulocyte-monocyte progenitor (GMP: (CD16/32+), common myeloid progenitor (CMP: CD16/32low) and megakaryocyte-erythrocyte progenitor (MEP: CD16/32−) – also do not differ between TNIKBWT and TNIKBKO mice (n = 19/20). (E) Number of mature immune (CD45+) cells, Plasma B cells (CD138+), CD4+ and CD8+ T cells (CD3+) in the bone marrow are not affected by TNIK B cell deficiency (n = 9/10). [file Image7.tif]

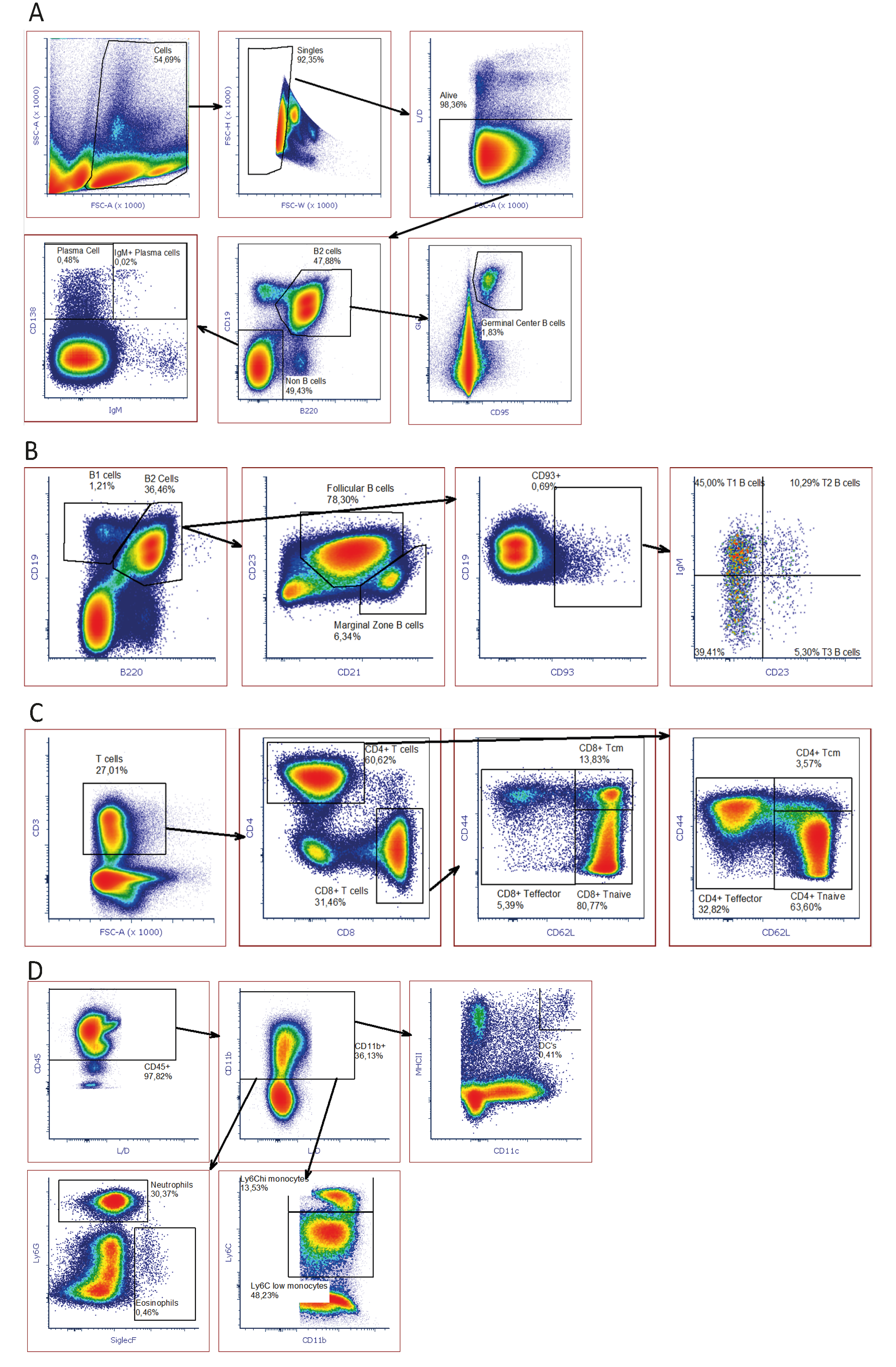

Supplement: Supplementary Figure 8 — Bone marrow differentiation unaffected due to B cell TNIK deficiency. Flow cytometric identification of bone marrow hematopoietic stem cells from TNIKBWT and TNIKBKO mice. (A) Total number of hematopoietic stem cells (Lineage−Sca-1+c-Kit+) showed a trend towards a decrease in TNIKBKO mice (n = 19/20). (B) Characterization of total number of long term stem cell (LT-SC: Lineage−Sca-1+c-Kit+CD150+CD48−), short term stem cell (ST-SC: Lineage−Sca-1+c-Kit+CD150−CD48−) showed no differences between genotypes (n = 19/20). Furthermore, no difference in multipotent progenitor cells (MPP: Sca-1+c-Kit+) or MPP subpopulations (MPP1a: CD48−CD150−135+, MPP1b: CD48−CD150−135+, MPP2: CD48+CD150+, MPP3:CD48+CD150−CD135+, MPP4:CD48+CD150−CD135+) was observed (n = 19/20). (C) Common lymphoid progenitors (CLP: Lineage−Sca-1lowc-Kit+CD48+CD16/CD32−) – early common lymphoid progenitor (CD127+CD135+), CLP (CD127+CD135−CD27−) and late CLP (CD127+CD135−CD27+) – did not reveal differences between TNIKBWT and TNIKBKO mice (n = 19/20). (D) Myeloid progenitors (Sca-1−cKit+) – granulocyte-monocyte progenitor (GMP: (CD16/32+), common myeloid progenitor (CMP: CD16/32low) and megakaryocyte-erythrocyte progenitor (MEP: CD16/32−) – also do not differ between TNIKBWT and TNIKBKO mice (n = 19/20). (E) Number of mature immune (CD45+) cells, Plasma B cells (CD138+), CD4+ and CD8+ T cells (CD3+) in the bone marrow are not affected by TNIK B cell deficiency (n = 9/10). [file Image8.tiff]

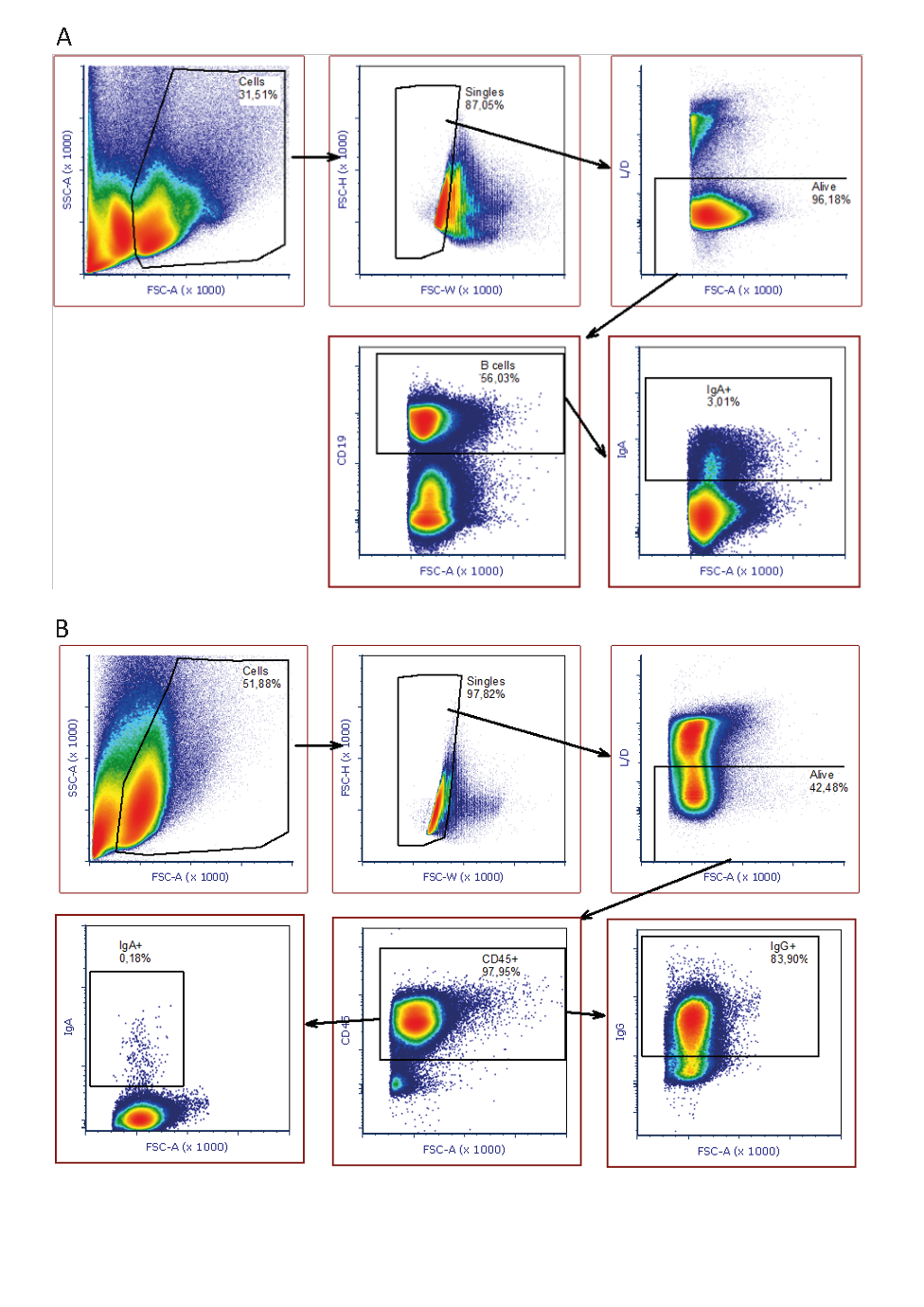

Supplement: Supplementary Method 1 — Gating strategy for flow cytometry on spleen, LN and blood samples. First three gating steps seen in figure (A) are applied to all other gating strategies. (A) Gating strategy for germinal center plasma cells. (B) Gating strategy for B cell differentiation subsets. (C) Gating strategy for CD4+ and CD8+ T cell subsets. (D) Gating strategy for myeloid cell populations. [file Image9.tif]

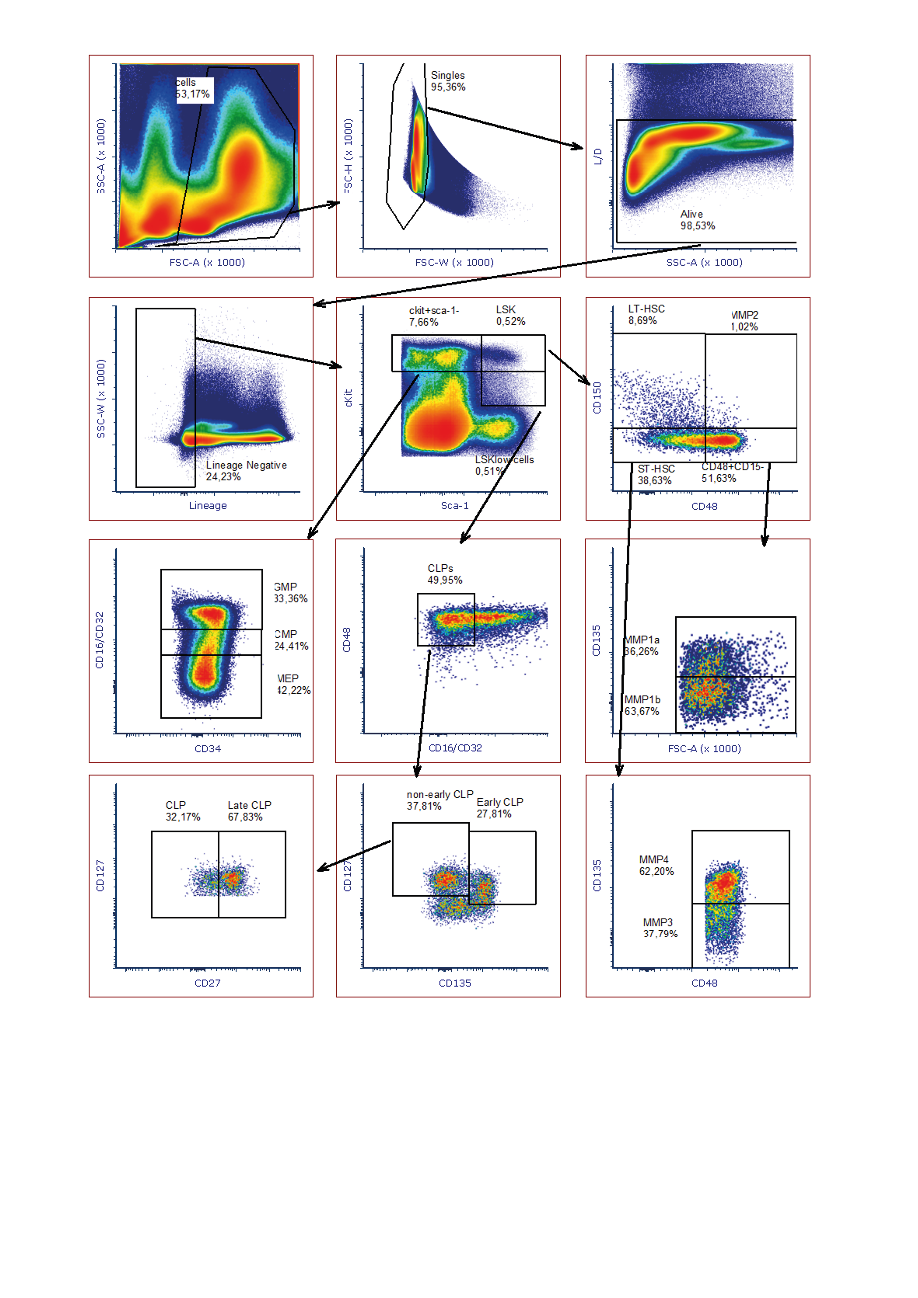

Supplement: Supplementary Method 2 — Gating strategy for flow cytometry on Peyer’s patches and spleen. (A) Gating strategy for IgA+ cells in Peyer’s Patches and spleen. (B) Gating strategy for IgM+ and IgG+ plasma cells cultured in vitro. [file Image10.tif]

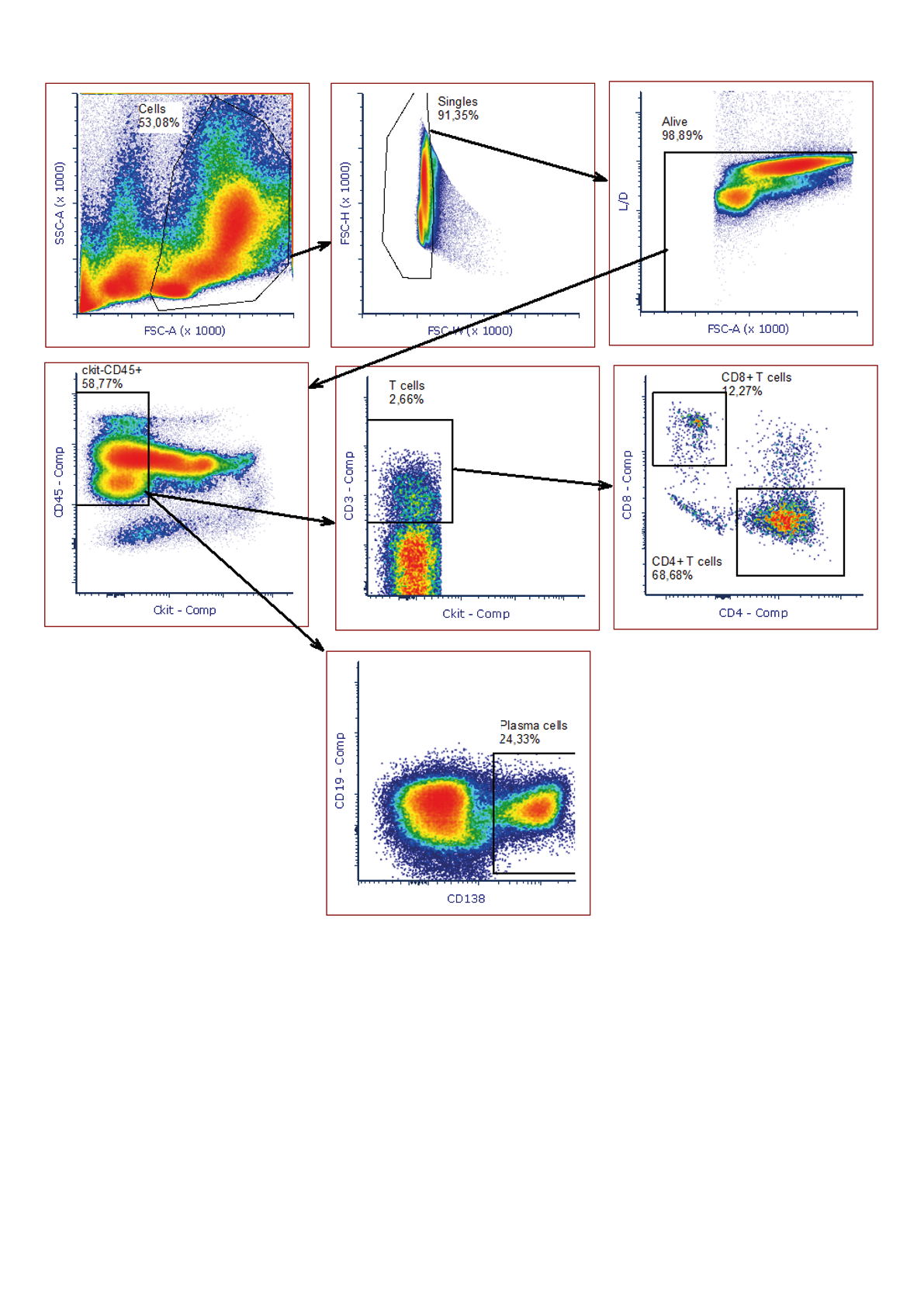

Supplement: Supplementary Method 3 — Gating strategy for stem cells. Gating strategy for stem cells from bone marrow samples. [file Image11.tif]
